# Supplementary figures and images for: Designing Antibiotic Cycling Strategies by Determining and Understanding Local Adaptive Landscapes
Source: PLoS One. 2013 Feb 13;8(2):e56040. doi: 10.1371/journal.pone.0056040 (PMC3572165; doi:10.1371/journal.pone.0056040)

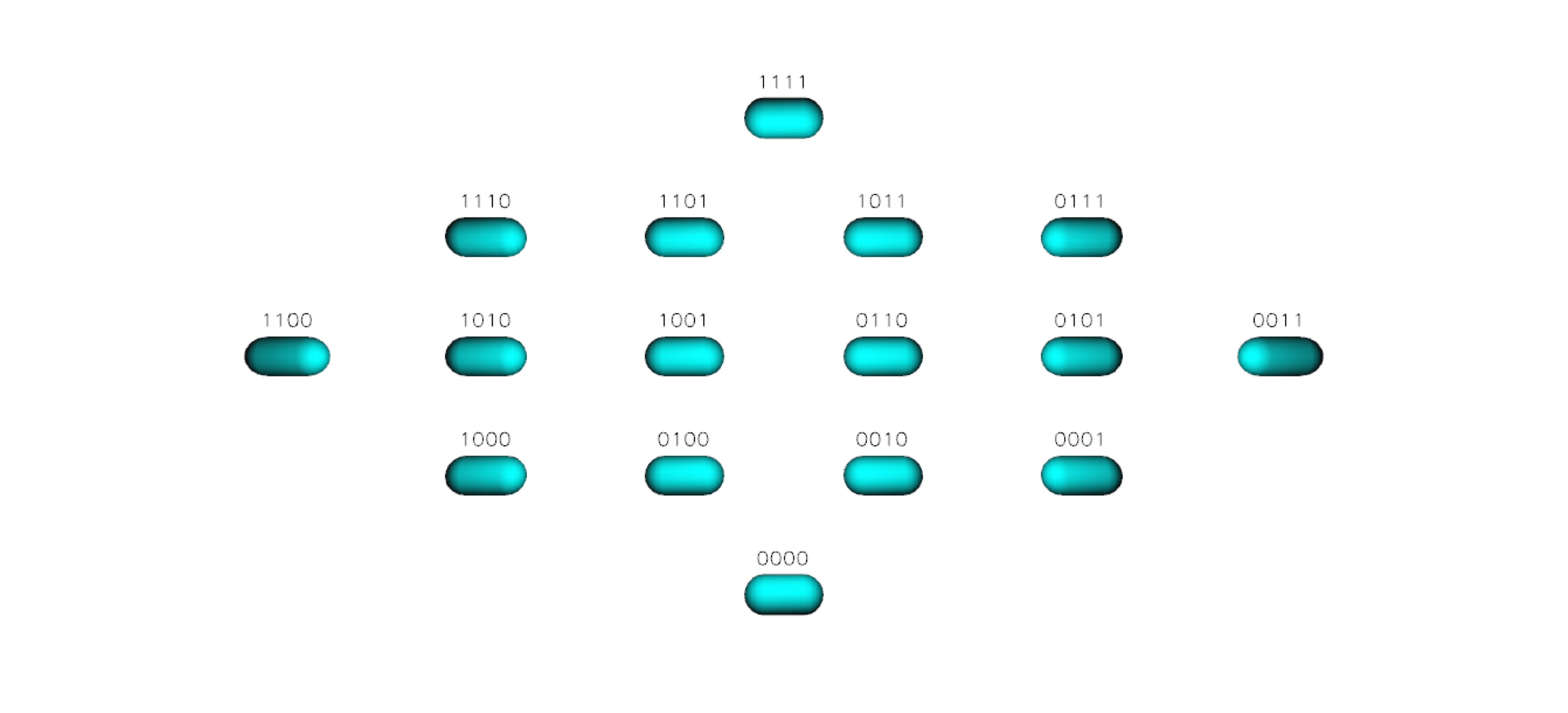


Figure S1 TEM 50 Landscape for Ampicillin

Supplement: Figure S1 — Figures of TEM-50 Adaptive Landscapes. Ovals represent alleles. The names are given in binary code (See table 1). The absence of lines indicates no significant difference in resistance phenotypes. Green lines indicate an increase in resistance resulting from addition of a mutation. Red lines indicate an increase in resistance resulting from reversion. (DOCX) [file pone.0056040.s001.docx]

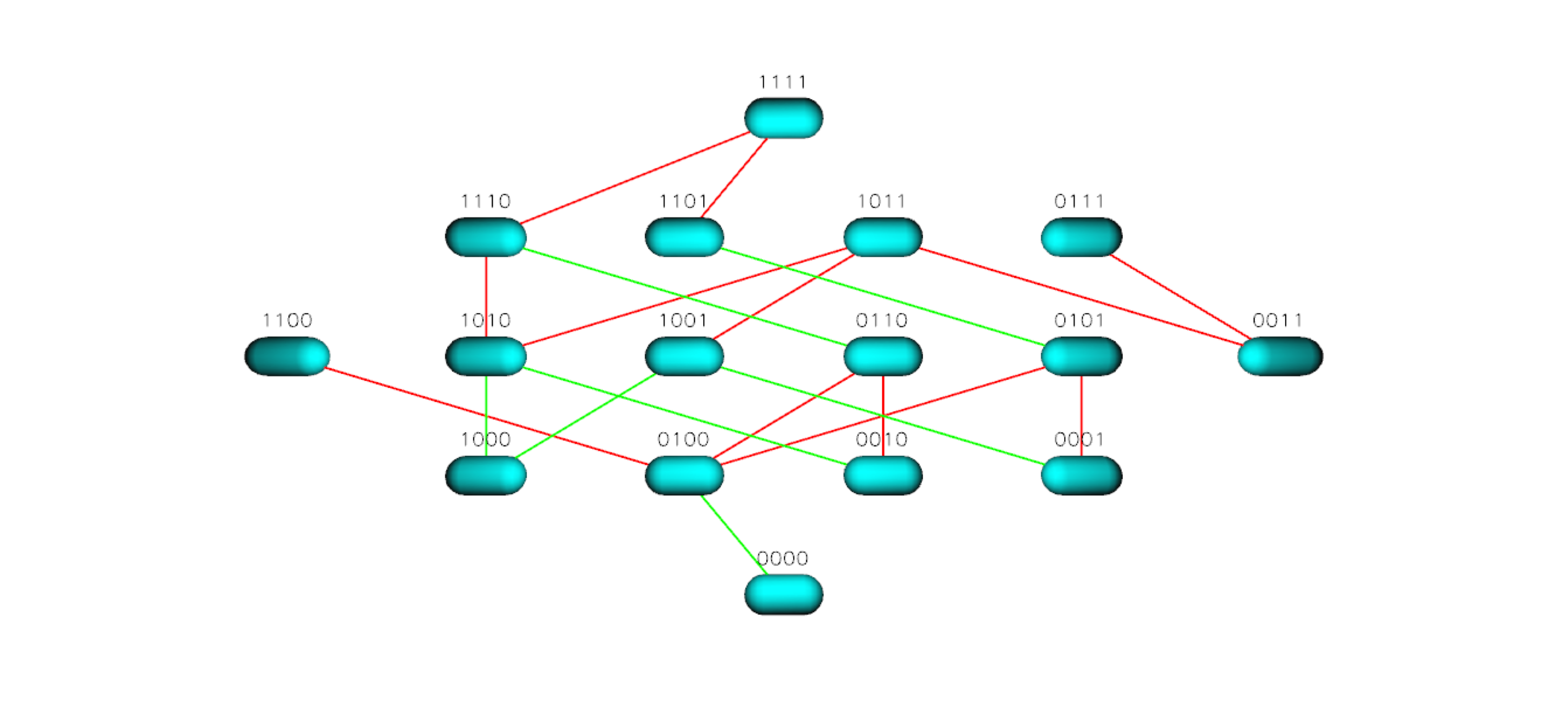


Figure S2 TEM 50 Landscape for Ceftazidime

Supplement: Figure S2 — Figures of TEM-50 Adaptive Landscapes. Ovals represent alleles. The names are given in binary code (See table 1). The absence of lines indicates no significant difference in resistance phenotypes. Green lines indicate an increase in resistance resulting from addition of a mutation. Red lines indicate an increase in resistance resulting from reversion. (DOCX) [file pone.0056040.s002.docx]

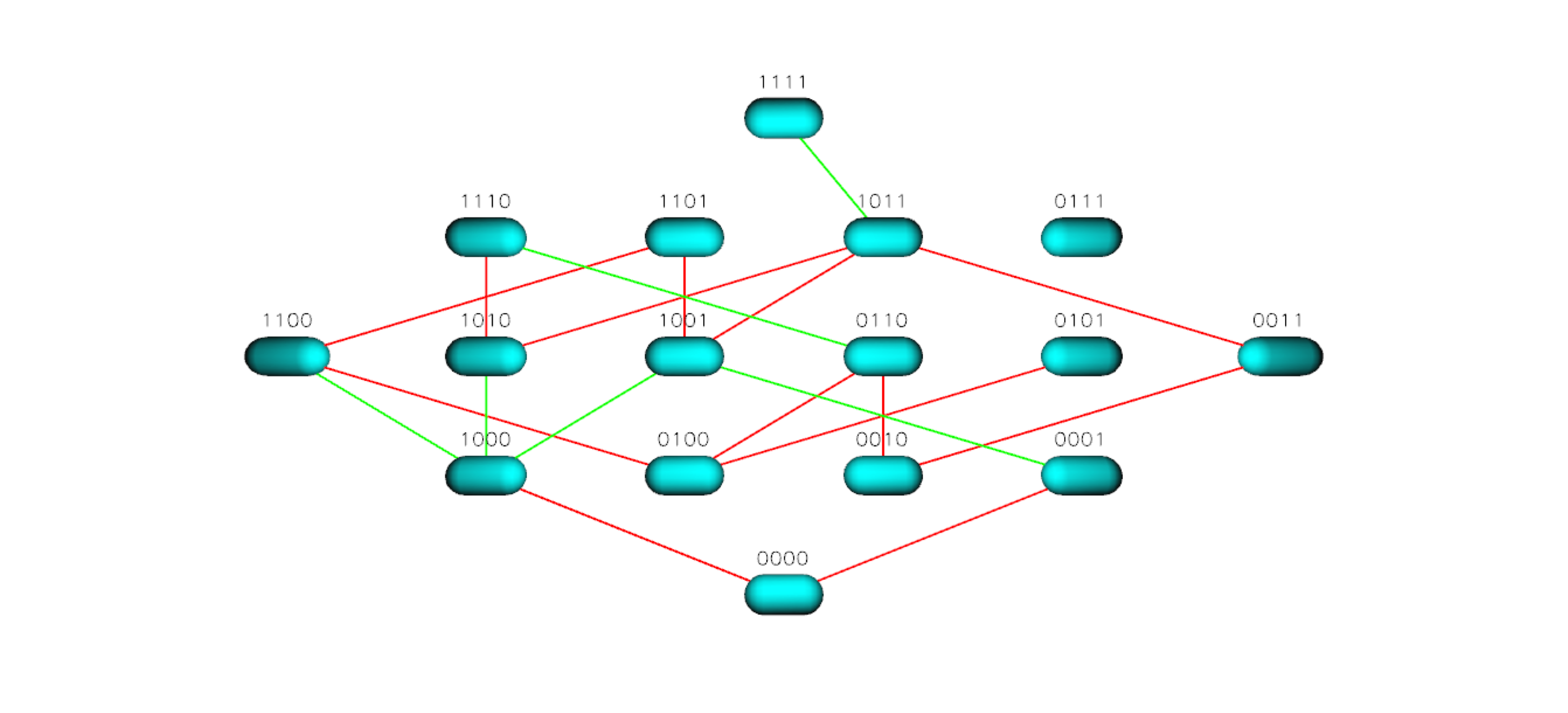


Figure S3 TEM 50 Landscape for Cefaclor

Supplement: Figure S3 — Figures of TEM-50 Adaptive Landscapes. Ovals represent alleles. The names are given in binary code (See table 1). The absence of lines indicates no significant difference in resistance phenotypes. Green lines indicate an increase in resistance resulting from addition of a mutation. Red lines indicate an increase in resistance resulting from reversion. (DOCX) [file pone.0056040.s003.docx]

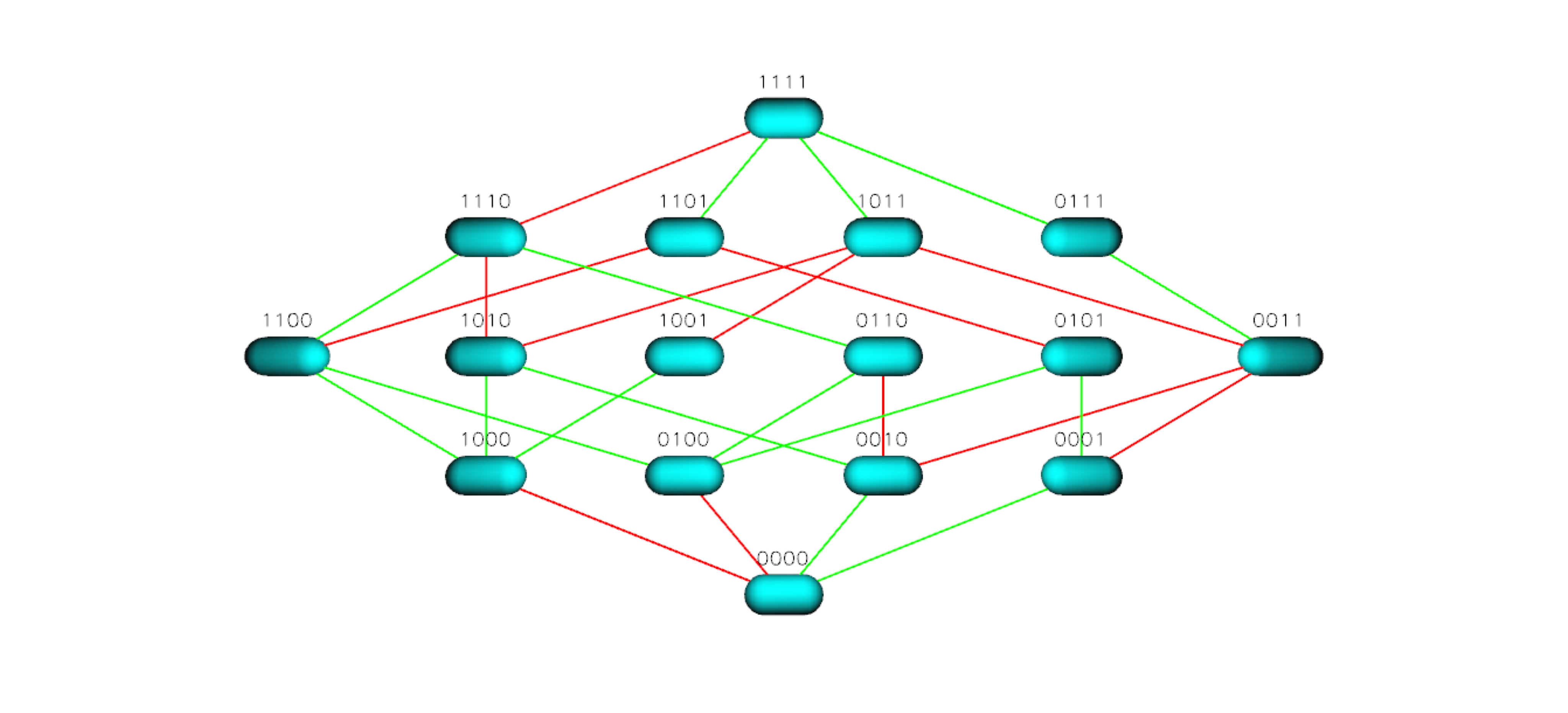


Figure S4 TEM 50 Landscape for Cefpodoxime

Supplement: Figure S4 — Figures of TEM-50 Adaptive Landscapes. Ovals represent alleles. The names are given in binary code (See table 1). The absence of lines indicates no significant difference in resistance phenotypes. Green lines indicate an increase in resistance resulting from addition of a mutation. Red lines indicate an increase in resistance resulting from reversion. (DOCX) [file pone.0056040.s004.docx]

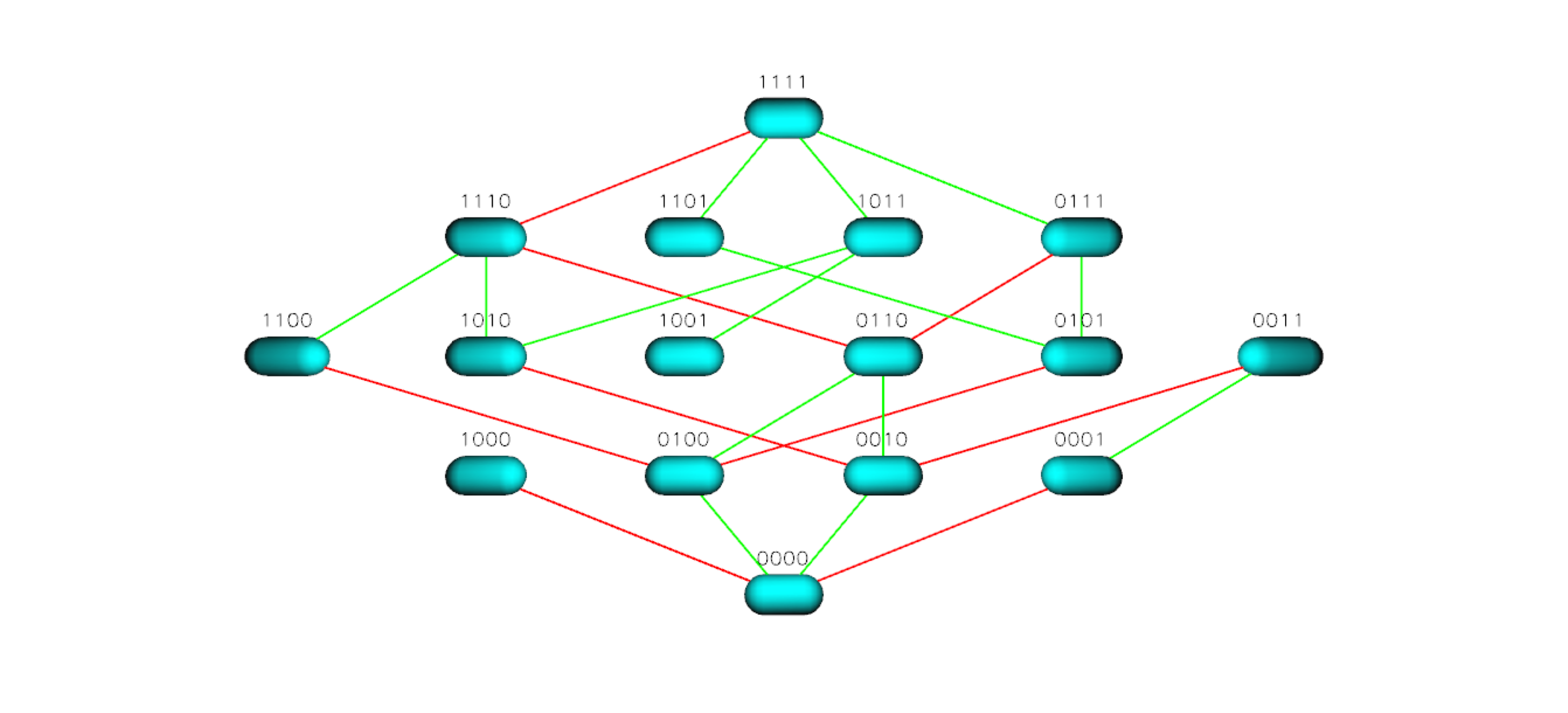


Figure S5 TEM 50 Landscape for Ceftriaxone

Supplement: Figure S5 — Figures of TEM-50 Adaptive Landscapes. Ovals represent alleles. The names are given in binary code (See table 1). The absence of lines indicates no significant difference in resistance phenotypes. Green lines indicate an increase in resistance resulting from addition of a mutation. Red lines indicate an increase in resistance resulting from reversion. (DOCX) [file pone.0056040.s005.docx]

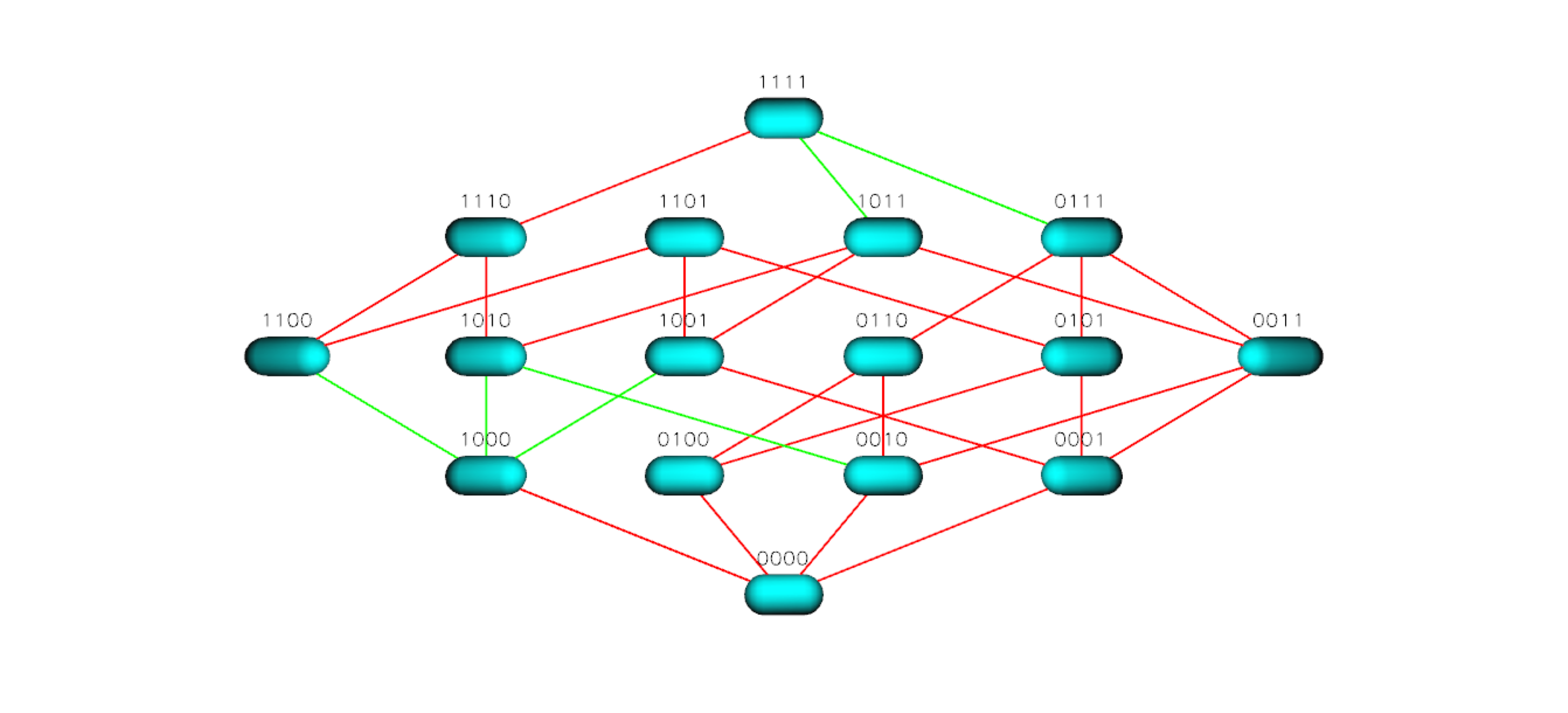


Figure S6 TEM 50 Landscape for Cefprozil

Supplement: Figure S6 — Figures of TEM-50 Adaptive Landscapes. Ovals represent alleles. The names are given in binary code (See table 1). The absence of lines indicates no significant difference in resistance phenotypes. Green lines indicate an increase in resistance resulting from addition of a mutation. Red lines indicate an increase in resistance resulting from reversion. (DOCX) [file pone.0056040.s006.docx]

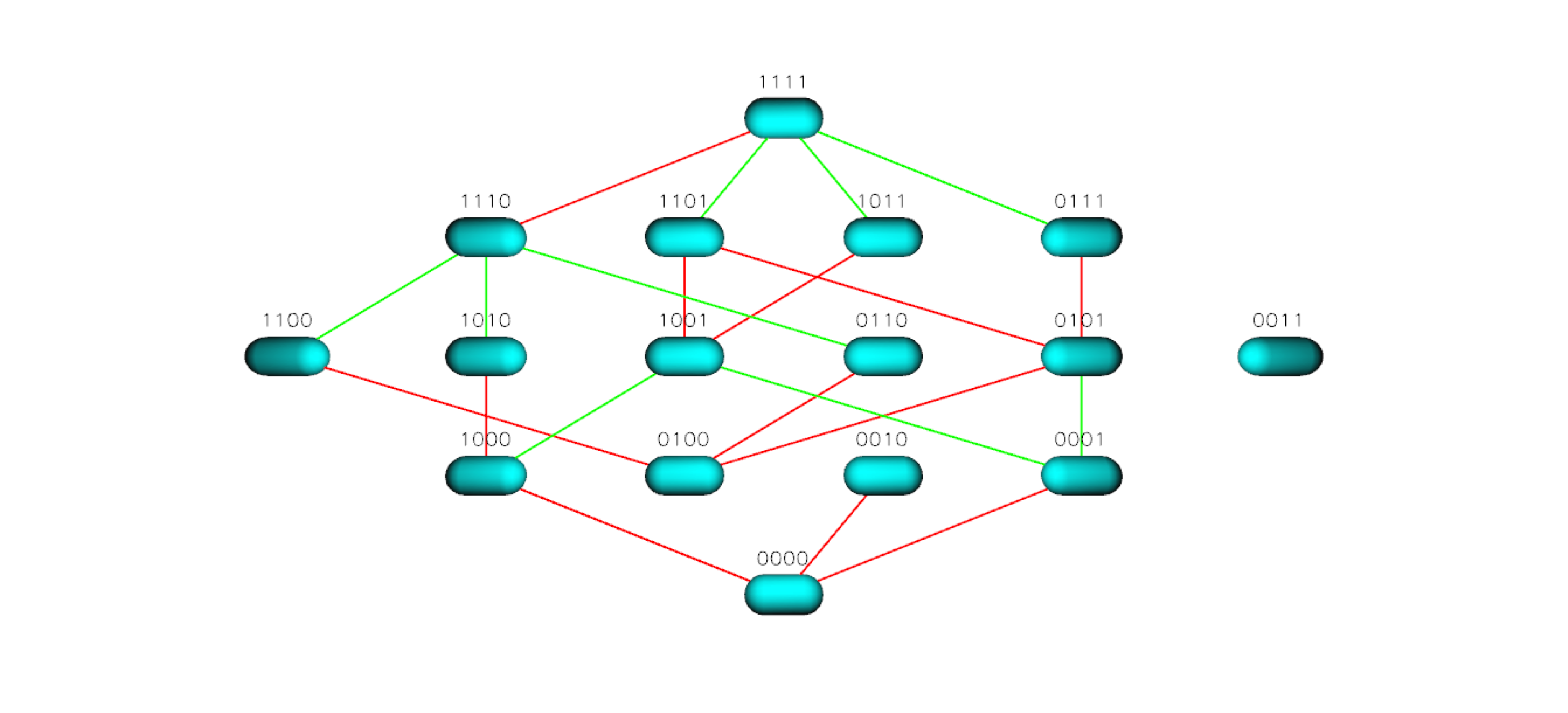


Figure S7 TEM 50 Landscape for Cefotetan

Supplement: Figure S7 — Figures of TEM-50 Adaptive Landscapes. Ovals represent alleles. The names are given in binary code (See table 1). The absence of lines indicates no significant difference in resistance phenotypes. Green lines indicate an increase in resistance resulting from addition of a mutation. Red lines indicate an increase in resistance resulting from reversion. (DOCX) [file pone.0056040.s007.docx]

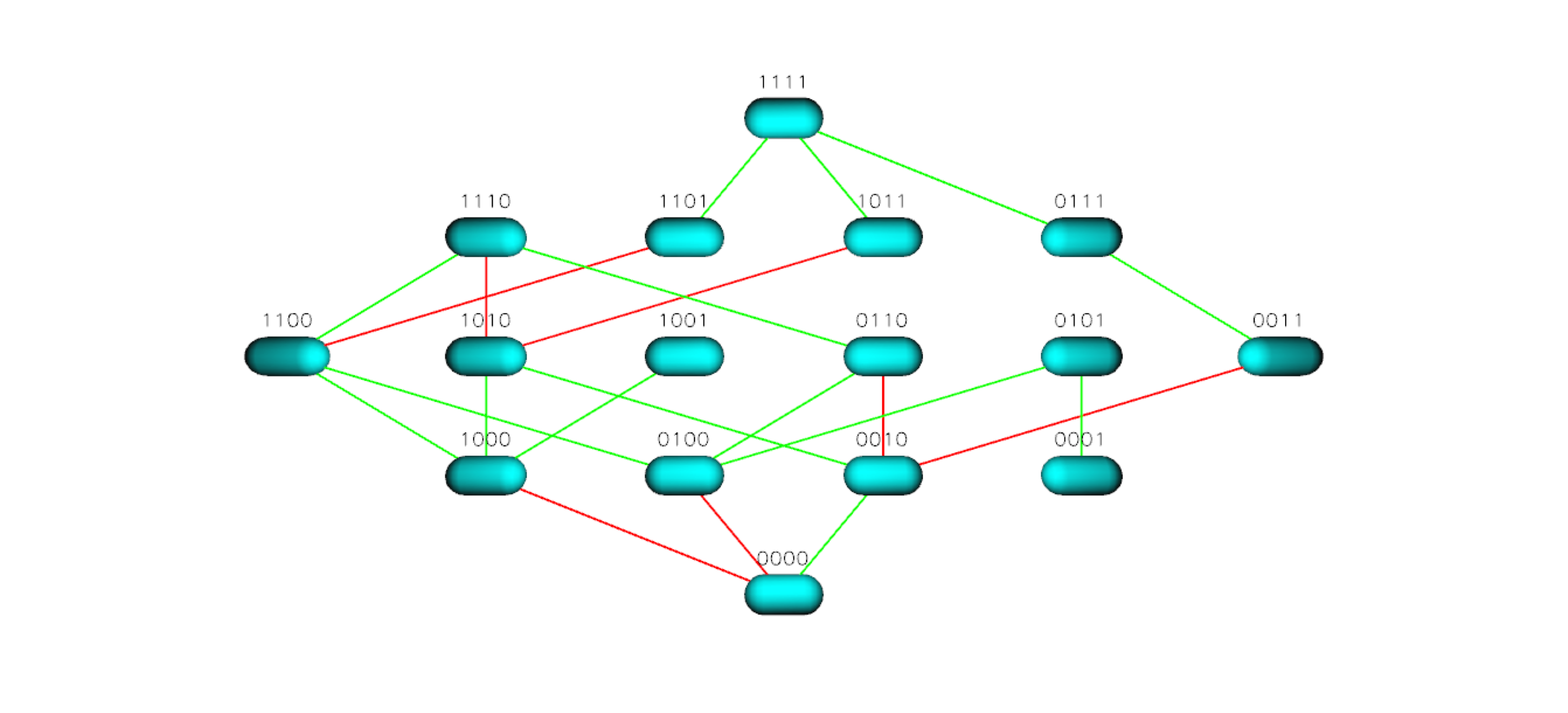


Figure S8 TEM 50 Landscape for Cefotaxime

Supplement: Figure S8 — Figures of TEM-50 Adaptive Landscapes. Ovals represent alleles. The names are given in binary code (See table 1). The absence of lines indicates no significant difference in resistance phenotypes. Green lines indicate an increase in resistance resulting from addition of a mutation. Red lines indicate an increase in resistance resulting from reversion. (DOCX) [file pone.0056040.s008.docx]

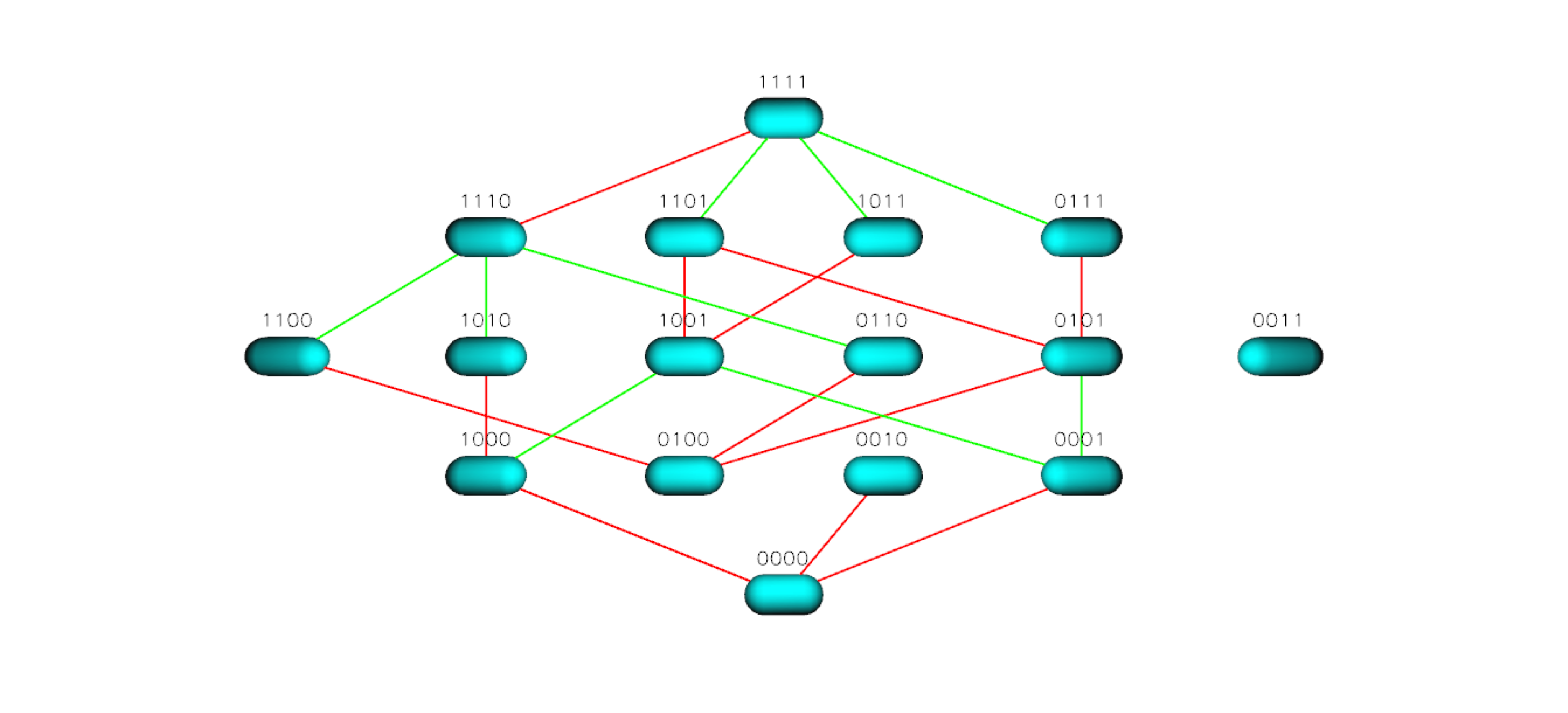


Figure S9 TEM 50 Landscape for Cefuroxime

Supplement: Figure S9 — Figures of TEM-50 Adaptive Landscapes. Ovals represent alleles. The names are given in binary code (See table 1). The absence of lines indicates no significant difference in resistance phenotypes. Green lines indicate an increase in resistance resulting from addition of a mutation. Red lines indicate an increase in resistance resulting from reversion. (DOCX) [file pone.0056040.s009.docx]

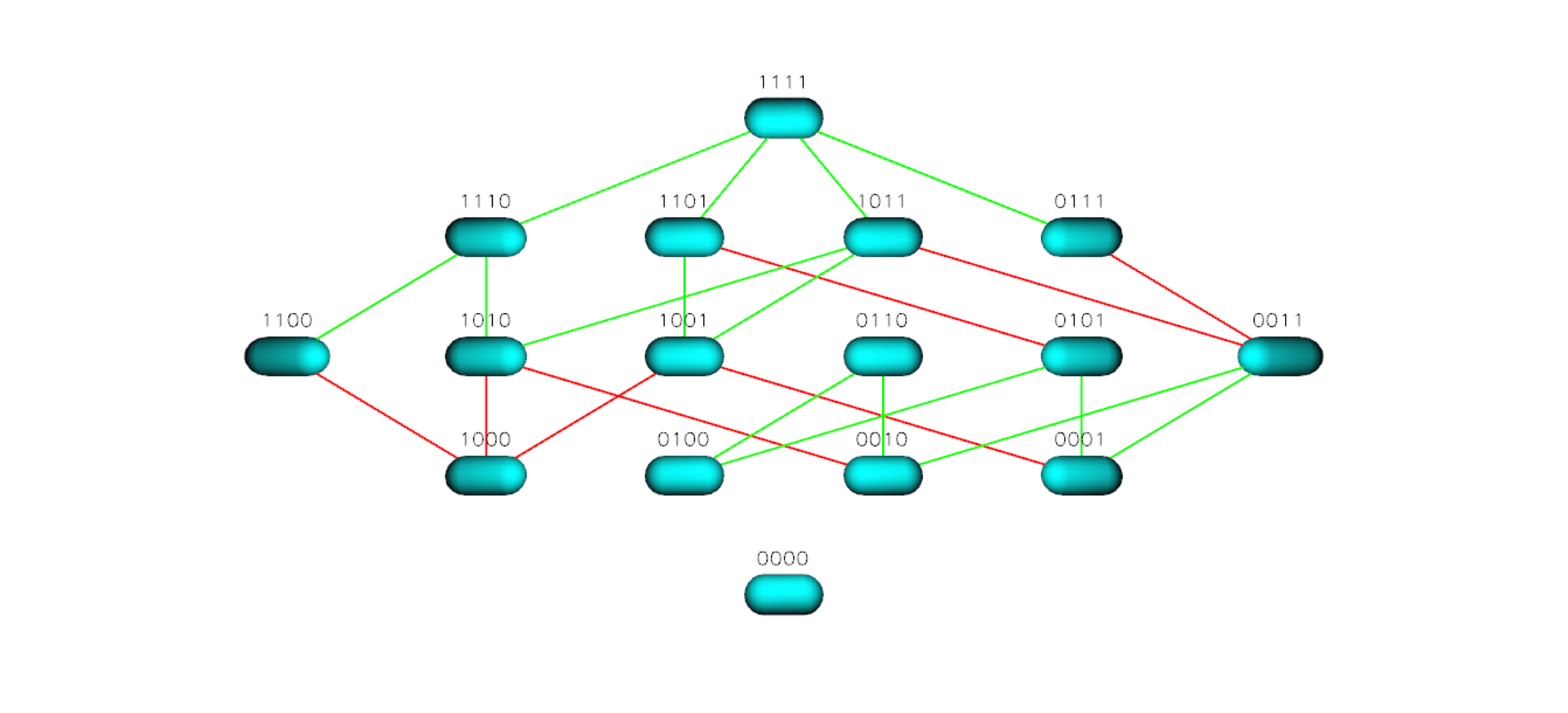


Figure S10 TEM 50 Landscape for Cefepime

Supplement: Figure S10 — Figures of TEM-50 Adaptive Landscapes. Ovals represent alleles. The names are given in binary code (See table 1). The absence of lines indicates no significant difference in resistance phenotypes. Green lines indicate an increase in resistance resulting from addition of a mutation. Red lines indicate an increase in resistance resulting from reversion. (DOCX) [file pone.0056040.s010.docx]

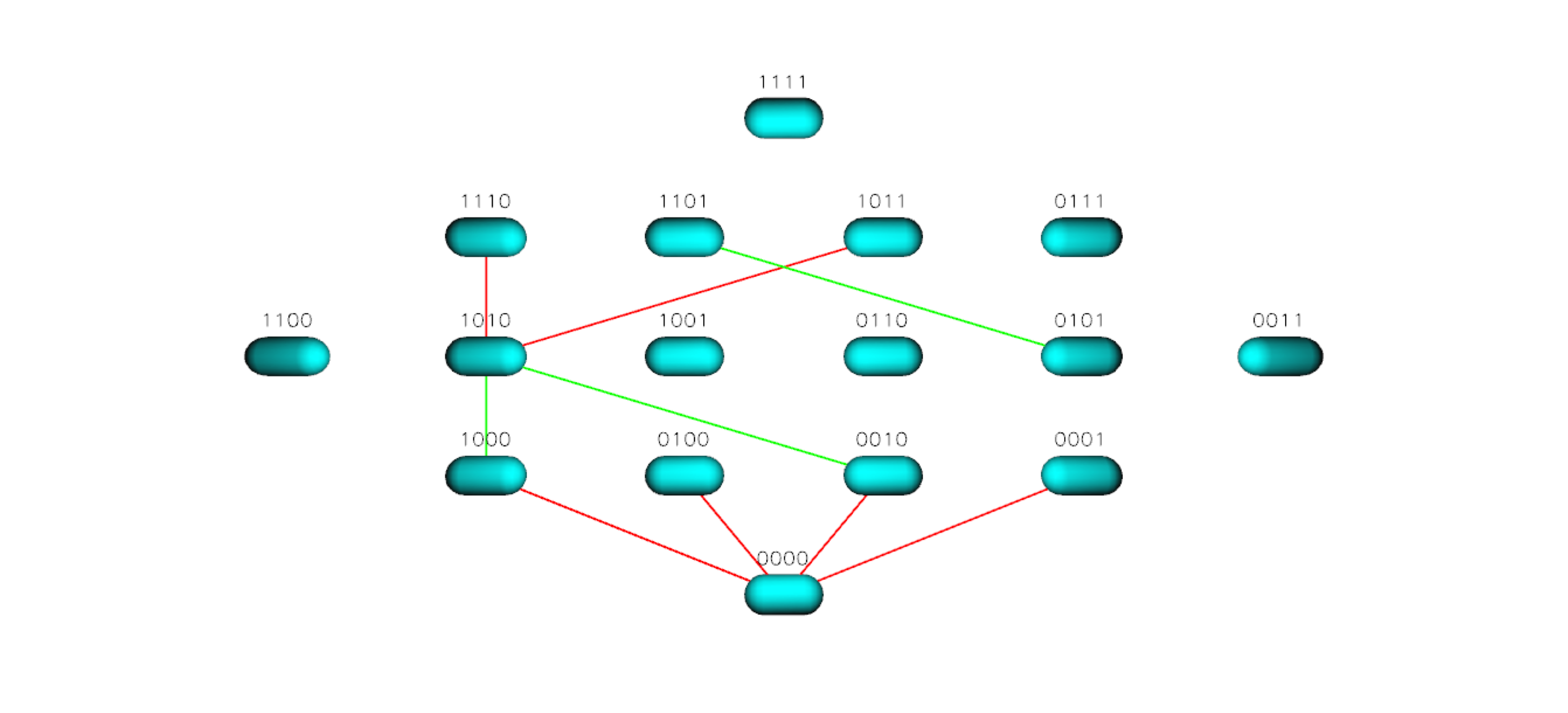


Figure S11 TEM 50 Landscape for Cefoxitin

Supplement: Figure S11 — Figures of TEM-50 Adaptive Landscapes. Ovals represent alleles. The names are given in binary code (See table 1). The absence of lines indicates no significant difference in resistance phenotypes. Green lines indicate an increase in resistance resulting from addition of a mutation. Red lines indicate an increase in resistance resulting from reversion. (DOCX) [file pone.0056040.s011.docx]

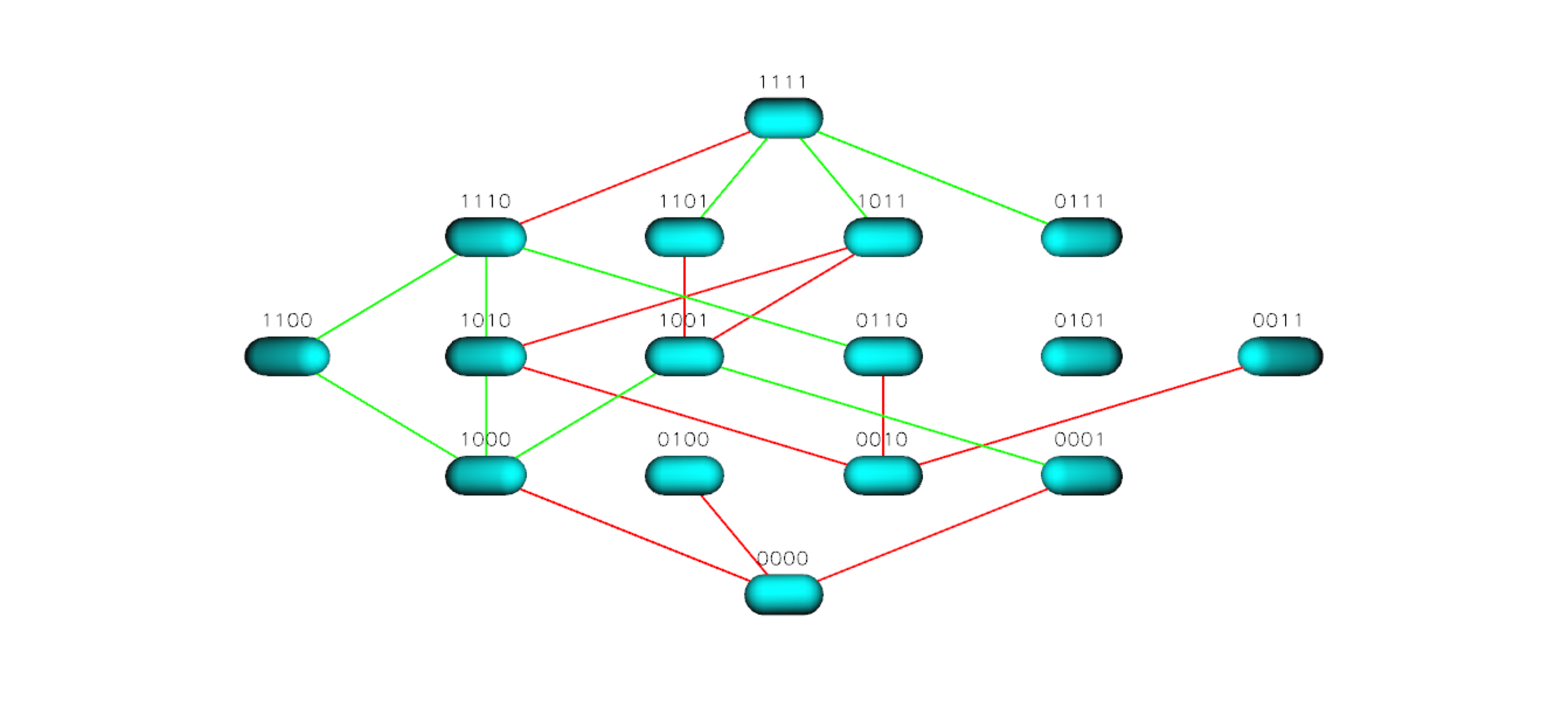


Figure S12 TEM 50 Landscape for Ceftizoxime

Supplement: Figure S12 — Figures of TEM-50 Adaptive Landscapes. Ovals represent alleles. The names are given in binary code (See table 1). The absence of lines indicates no significant difference in resistance phenotypes. Green lines indicate an increase in resistance resulting from addition of a mutation. Red lines indicate an increase in resistance resulting from reversion. (DOCX) [file pone.0056040.s012.docx]

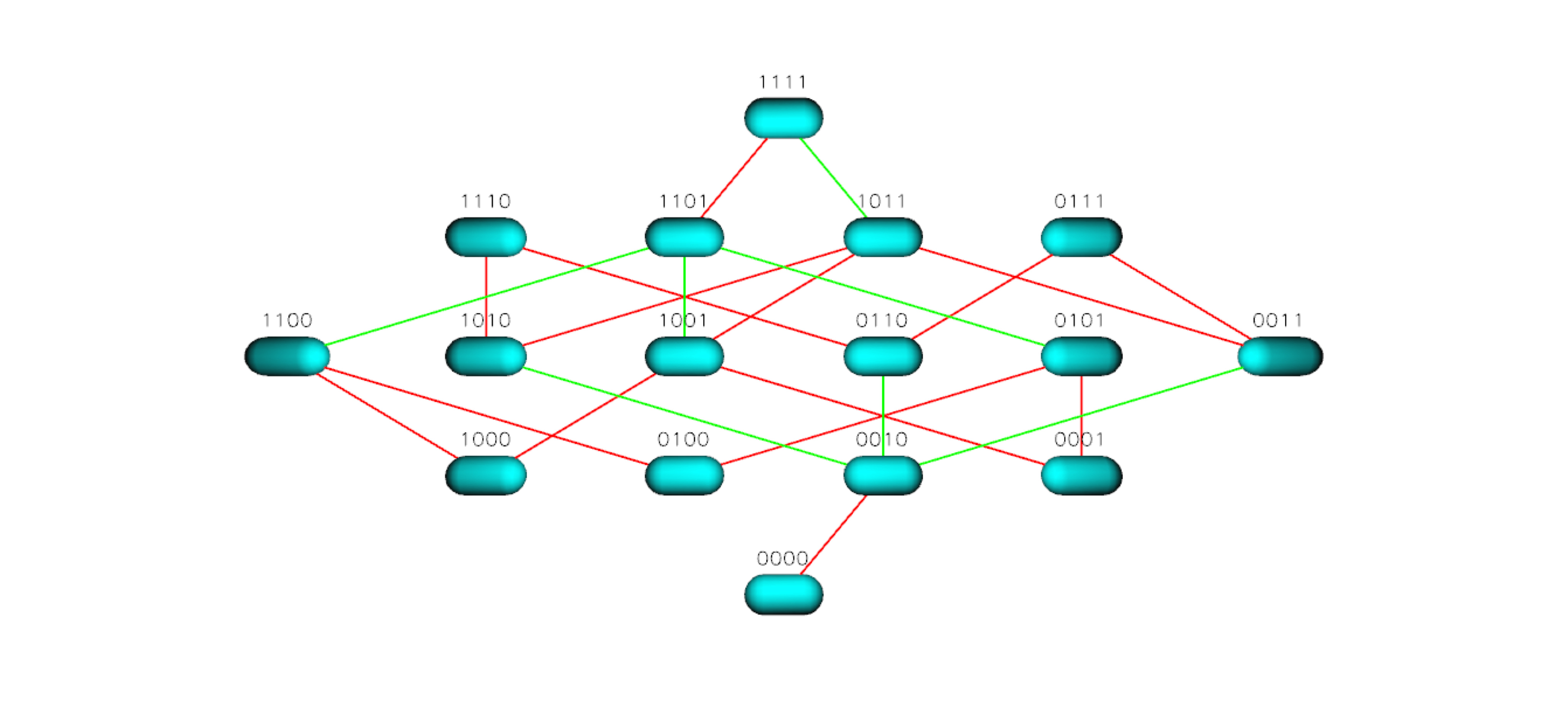


Figure S13 TEM 50 Landscape for Ampicillin + Sulbactam

Supplement: Figure S13 — Figures of TEM-50 Adaptive Landscapes. Ovals represent alleles. The names are given in binary code (See table 1). The absence of lines indicates no significant difference in resistance phenotypes. Green lines indicate an increase in resistance resulting from addition of a mutation. Red lines indicate an increase in resistance resulting from reversion. (DOCX) [file pone.0056040.s013.docx]

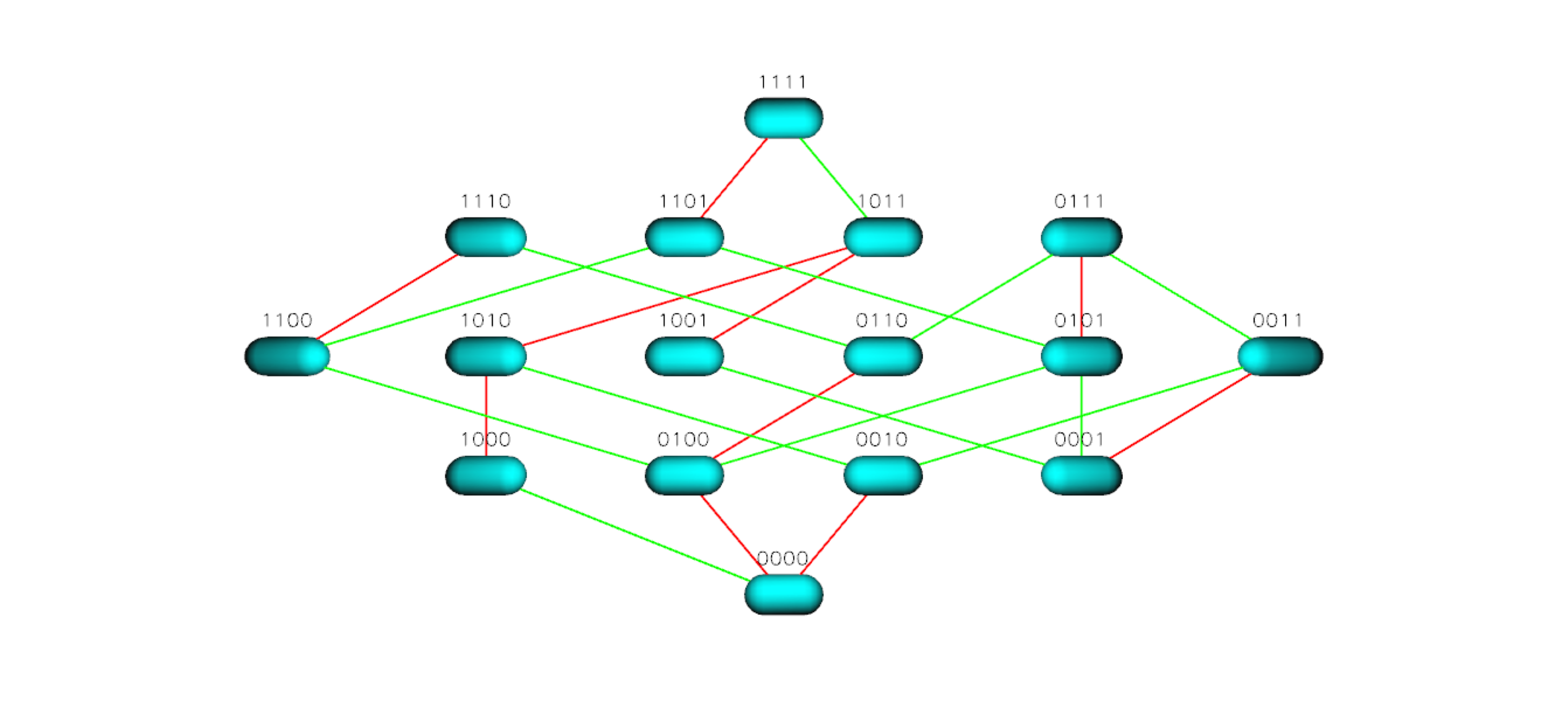


Figure S14 TEM 50 Landscape for Pipercillin + Tazobactam

Supplement: Figure S14 — Figures of TEM-50 Adaptive Landscapes. Ovals represent alleles. The names are given in binary code (See table 1). The absence of lines indicates no significant difference in resistance phenotypes. Green lines indicate an increase in resistance resulting from addition of a mutation. Red lines indicate an increase in resistance resulting from reversion. (DOCX) [file pone.0056040.s014.docx]

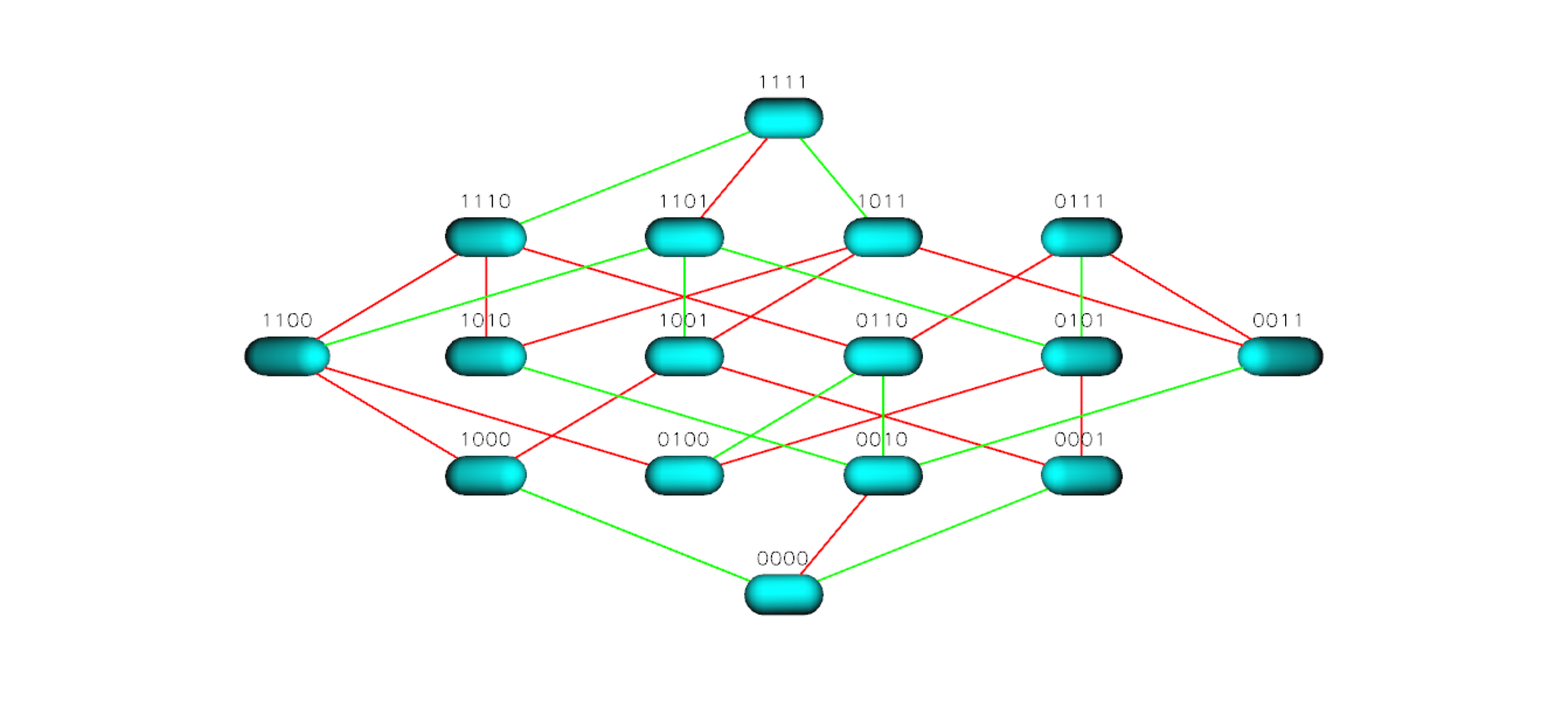


Figure S15 TEM 50 Landscape for Amoxicillin + Clavulanate

Supplement: Figure S15 — Figures of TEM-50 Adaptive Landscapes. Ovals represent alleles. The names are given in binary code (See table 1). The absence of lines indicates no significant difference in resistance phenotypes. Green lines indicate an increase in resistance resulting from addition of a mutation. Red lines indicate an increase in resistance resulting from reversion. (DOCX) [file pone.0056040.s015.docx]

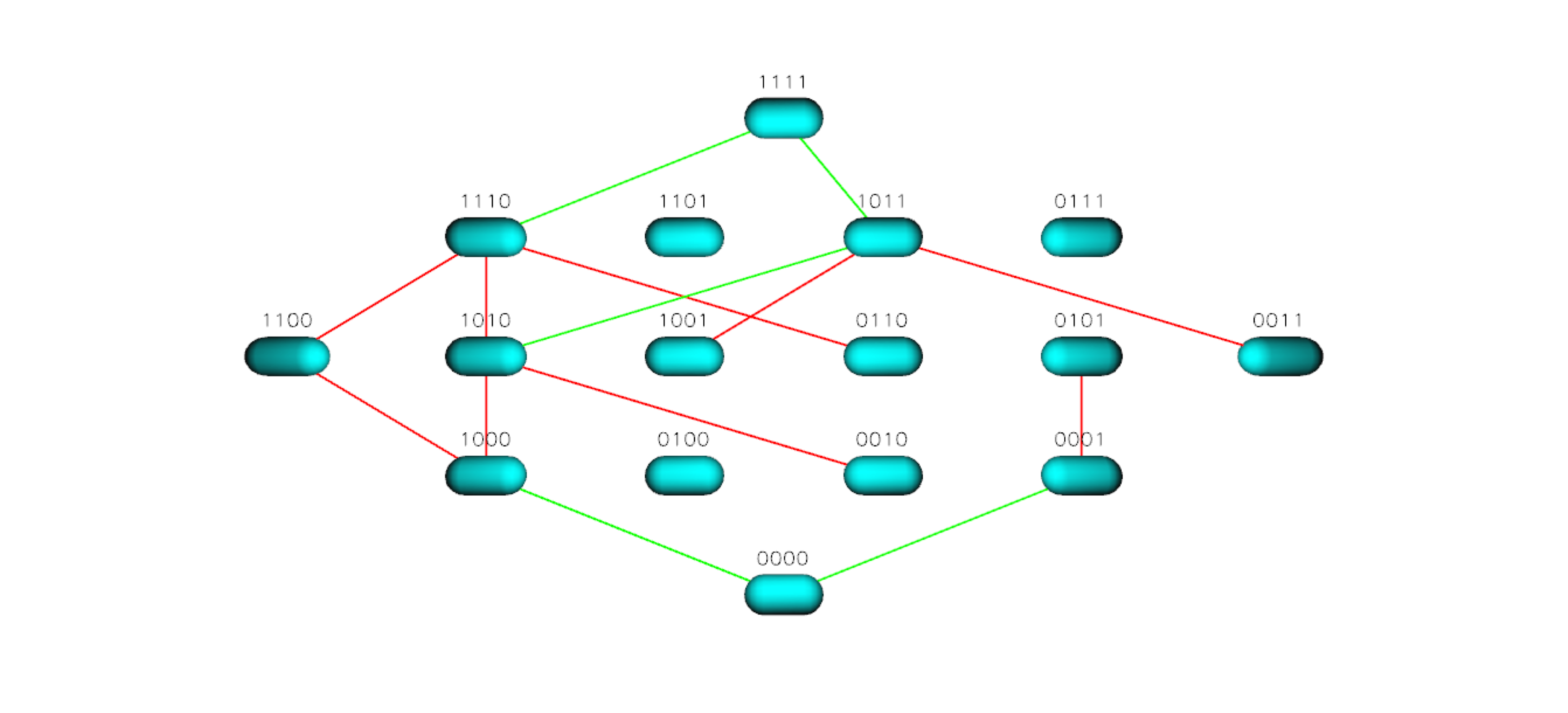


Figure S16 TEM 85 Landscape for Ampicillin

Supplement: Figure S16 — Figures of TEM-85 Adaptive Landscapes. Ovals represent alleles. The names are given in binary code (See table 1). The absence of lines indicates no significant difference in resistance phenotypes. Green lines indicate an increase in resistance resulting from addition of a mutation. Red lines indicate an increase in resistance resulting from reversion. (DOCX) [file pone.0056040.s016.docx]

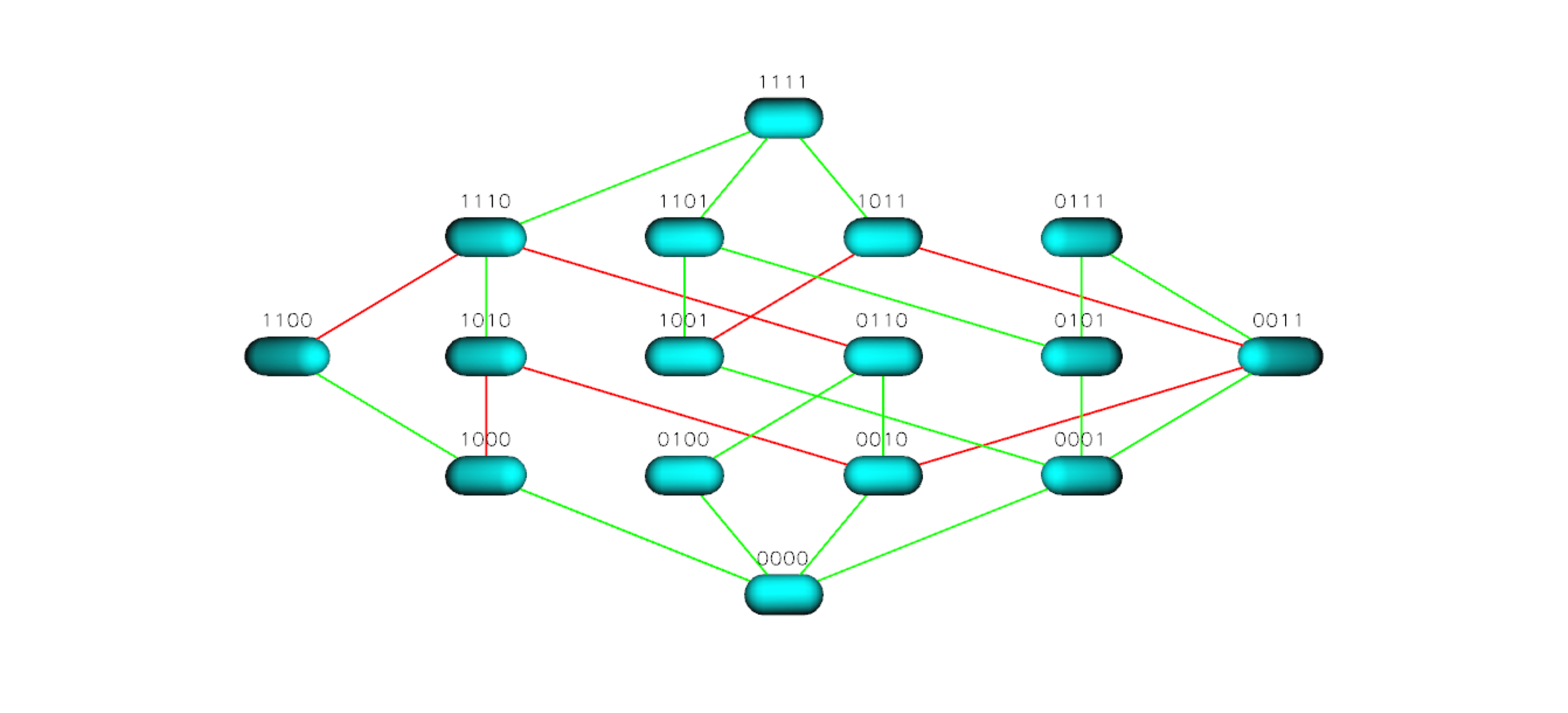


Figure S17 TEM 85 Landscape for Ceftazidime

Supplement: Figure S17 — Figures of TEM-85 Adaptive Landscapes. Ovals represent alleles. The names are given in binary code (See table 1). The absence of lines indicates no significant difference in resistance phenotypes. Green lines indicate an increase in resistance resulting from addition of a mutation. Red lines indicate an increase in resistance resulting from reversion. (DOCX) [file pone.0056040.s017.docx]

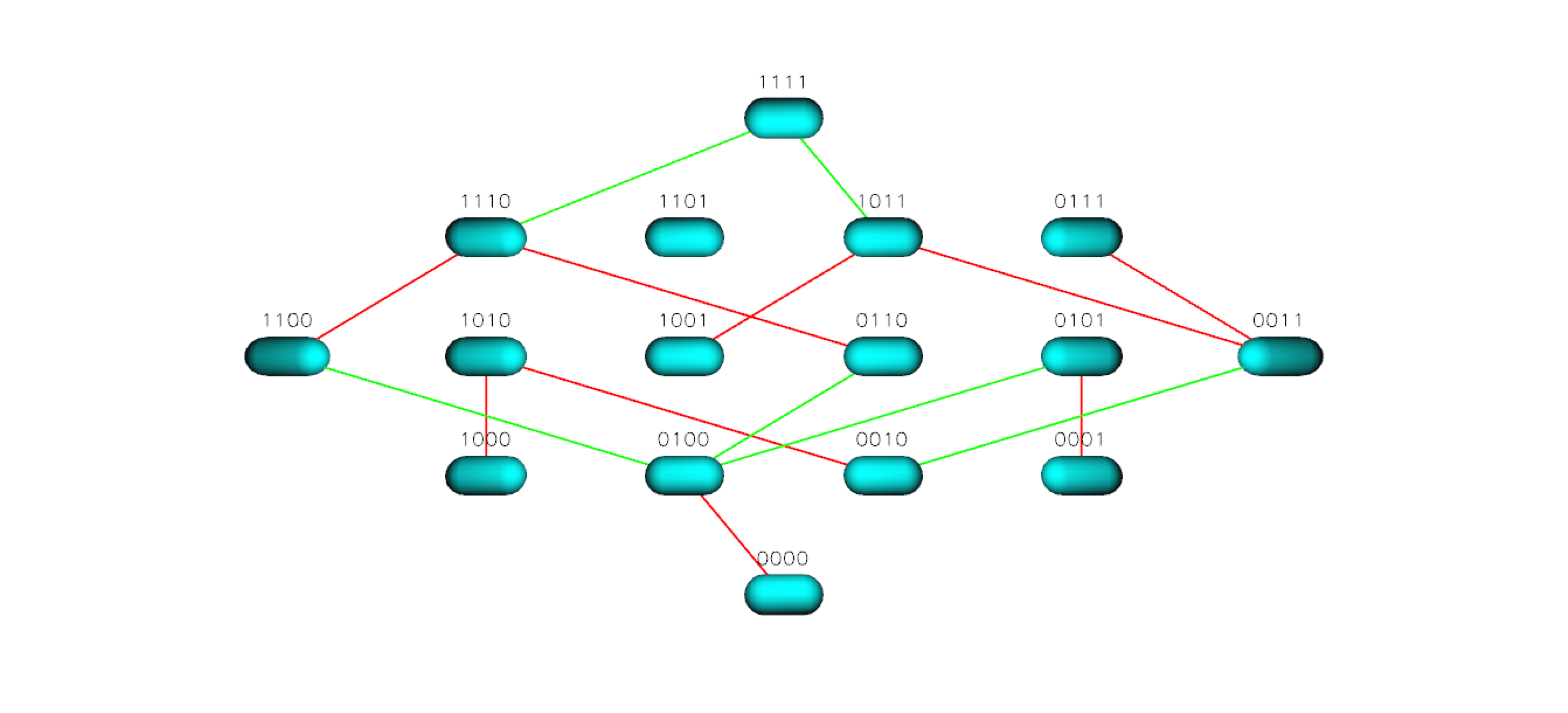


Figure S18 TEM 85 Landscape for Cefaclor

Supplement: Figure S18 — Figures of TEM-85 Adaptive Landscapes. Ovals represent alleles. The names are given in binary code (See table 1). The absence of lines indicates no significant difference in resistance phenotypes. Green lines indicate an increase in resistance resulting from addition of a mutation. Red lines indicate an increase in resistance resulting from reversion. (DOCX) [file pone.0056040.s018.docx]

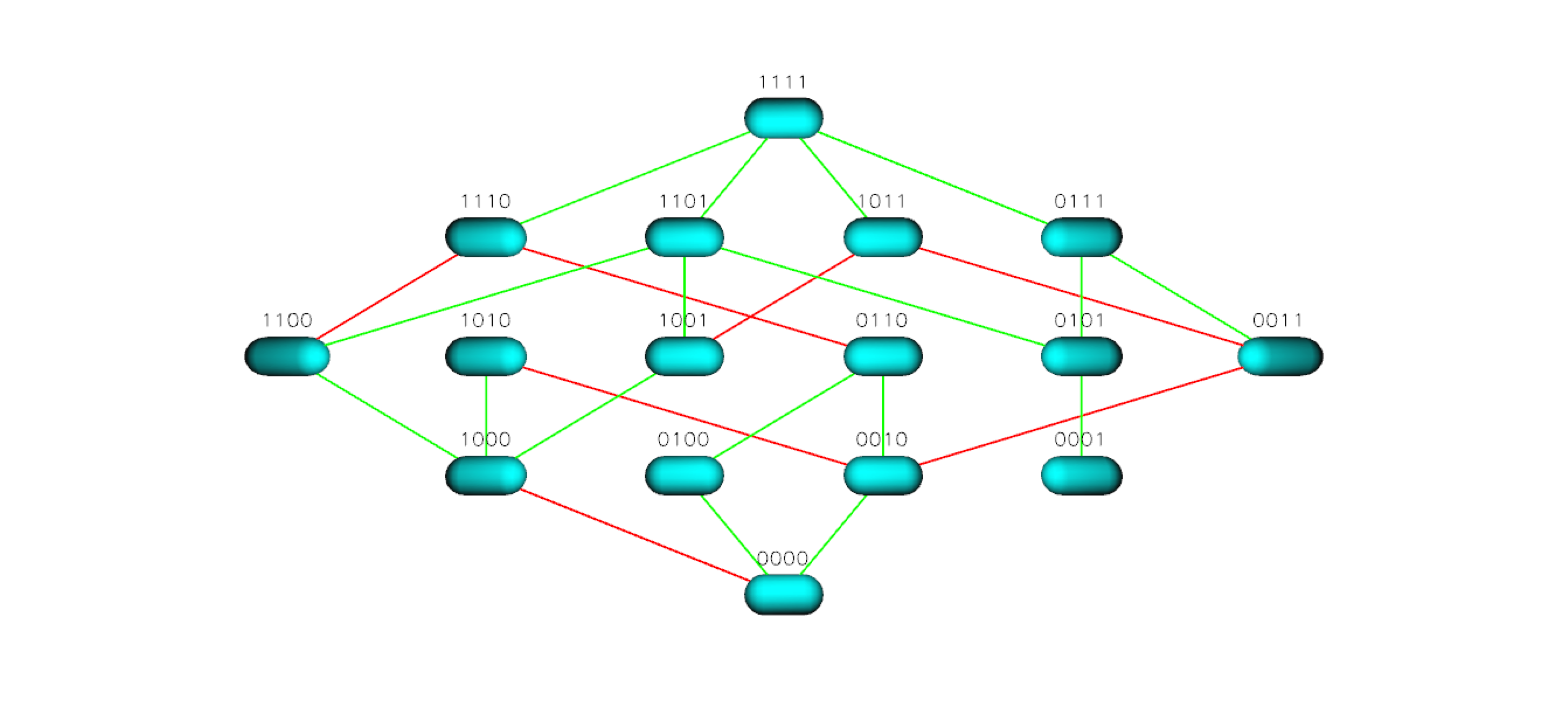


Figure S19 TEM 85 Landscape for Cefpodoxime

Supplement: Figure S19 — Figures of TEM-85 Adaptive Landscapes. Ovals represent alleles. The names are given in binary code (See table 1). The absence of lines indicates no significant difference in resistance phenotypes. Green lines indicate an increase in resistance resulting from addition of a mutation. Red lines indicate an increase in resistance resulting from reversion. (DOCX) [file pone.0056040.s019.docx]

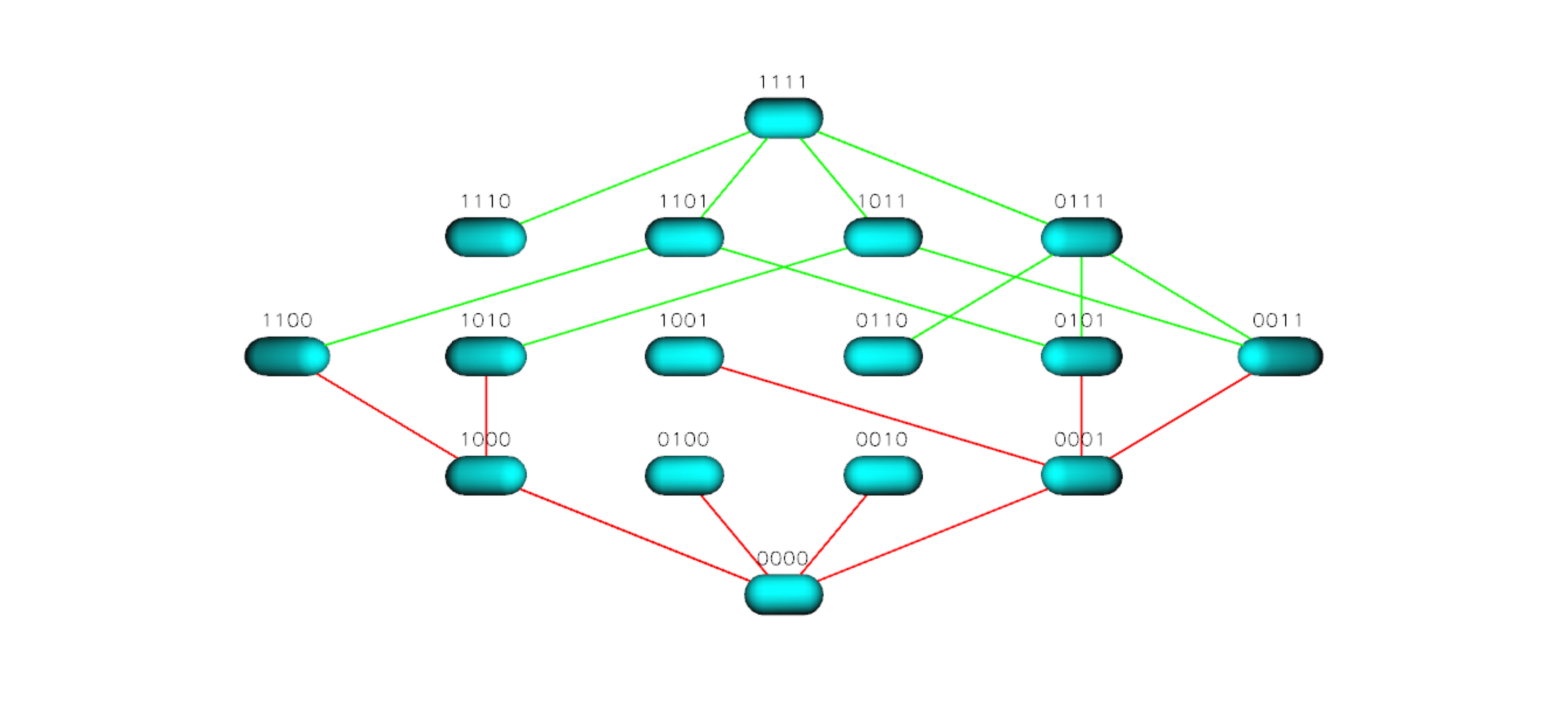


Figure S20 TEM 85 Landscape for Ceftriaxone

Supplement: Figure S20 — Figures of TEM-85 Adaptive Landscapes. Ovals represent alleles. The names are given in binary code (See table 1). The absence of lines indicates no significant difference in resistance phenotypes. Green lines indicate an increase in resistance resulting from addition of a mutation. Red lines indicate an increase in resistance resulting from reversion. (DOCX) [file pone.0056040.s020.docx]

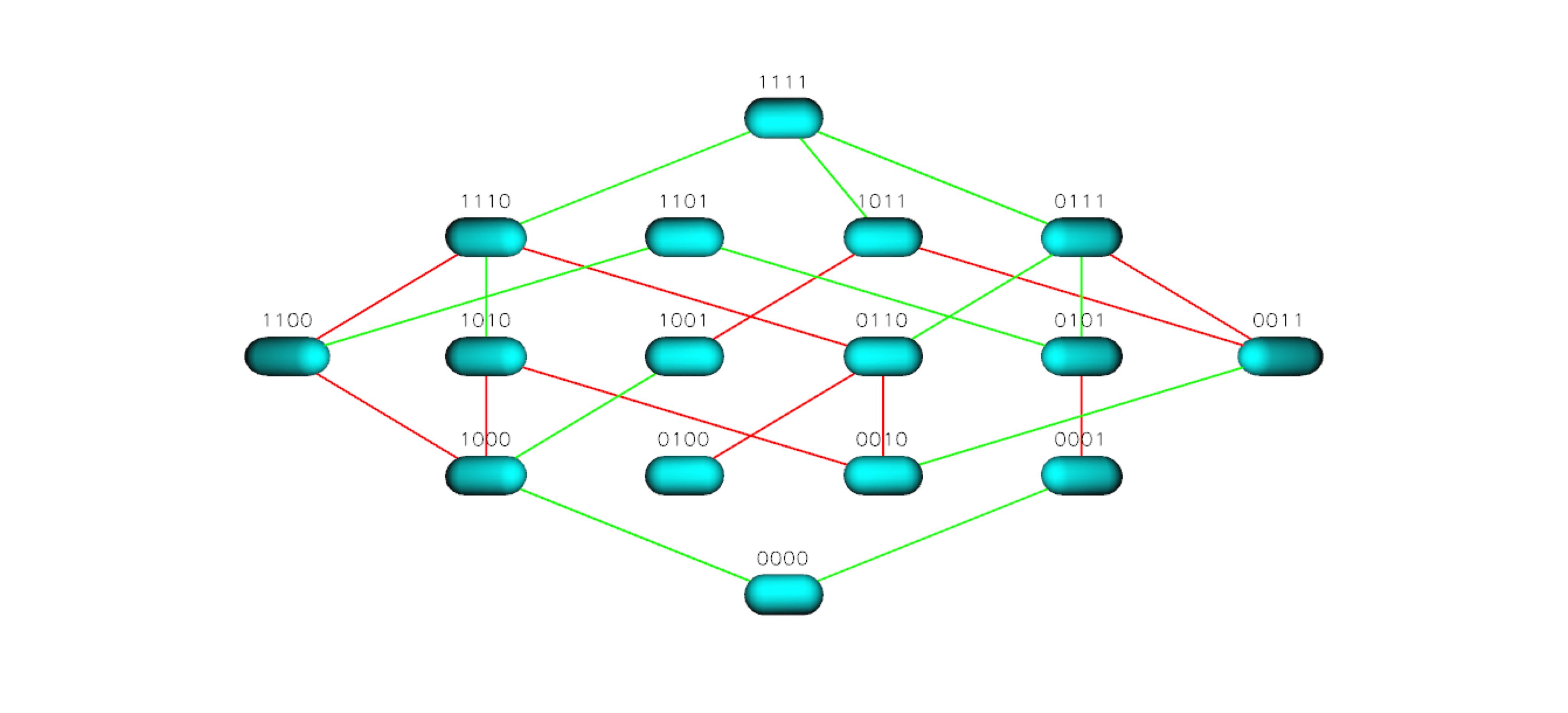


Figure S21 TEM 85 Landscape for Cefprozil

Supplement: Figure S21 — Figures of TEM-85 Adaptive Landscapes. Ovals represent alleles. The names are given in binary code (See table 1). The absence of lines indicates no significant difference in resistance phenotypes. Green lines indicate an increase in resistance resulting from addition of a mutation. Red lines indicate an increase in resistance resulting from reversion. (DOCX) [file pone.0056040.s021.docx]

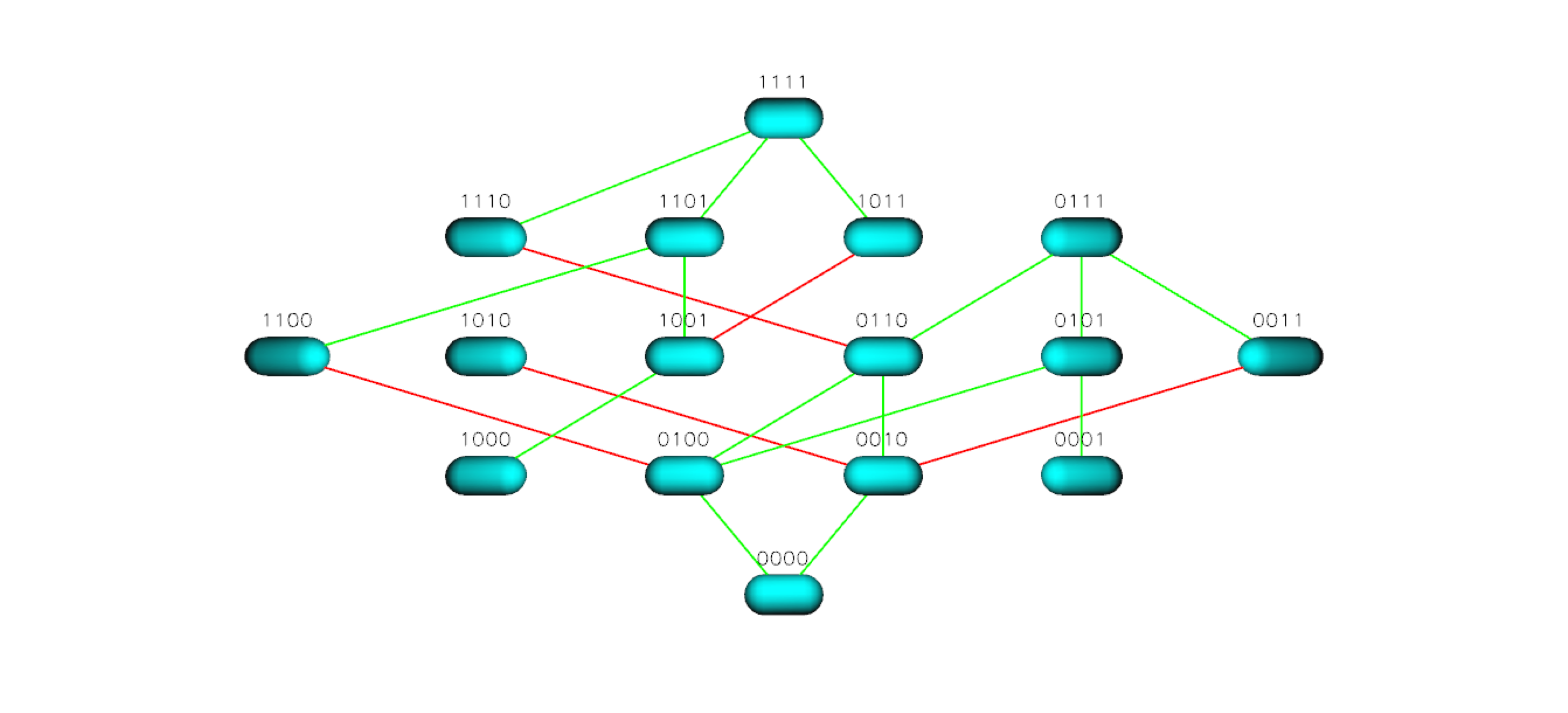


Figure S22 TEM 85 Landscape for Cefotetan

Supplement: Figure S22 — Figures of TEM-85 Adaptive Landscapes. Ovals represent alleles. The names are given in binary code (See table 1). The absence of lines indicates no significant difference in resistance phenotypes. Green lines indicate an increase in resistance resulting from addition of a mutation. Red lines indicate an increase in resistance resulting from reversion. (DOCX) [file pone.0056040.s022.docx]

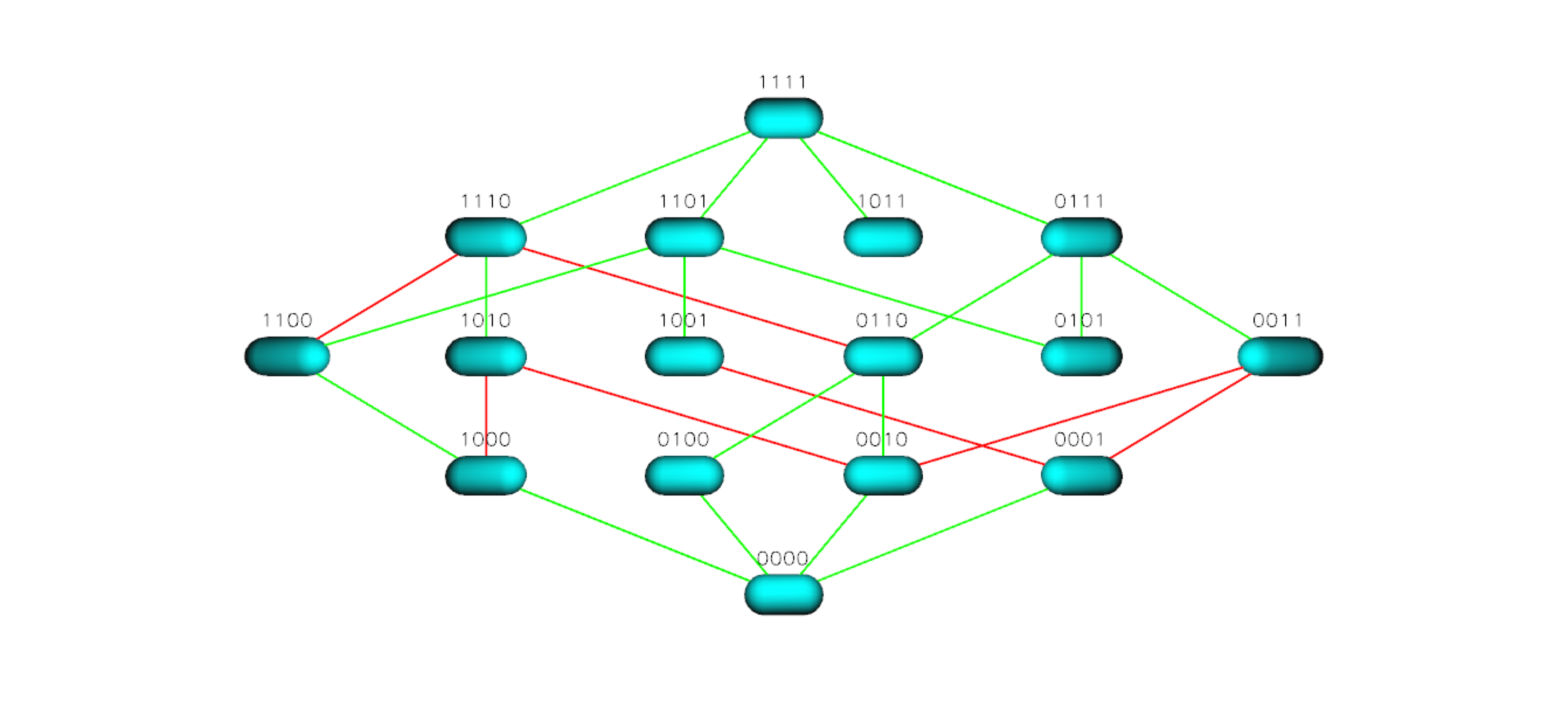


Figure S23 TEM 85 Landscape for Cefotaxime

Supplement: Figure S23 — Figures of TEM-85 Adaptive Landscapes. Ovals represent alleles. The names are given in binary code (See table 1). The absence of lines indicates no significant difference in resistance phenotypes. Green lines indicate an increase in resistance resulting from addition of a mutation. Red lines indicate an increase in resistance resulting from reversion. (DOCX) [file pone.0056040.s023.docx]

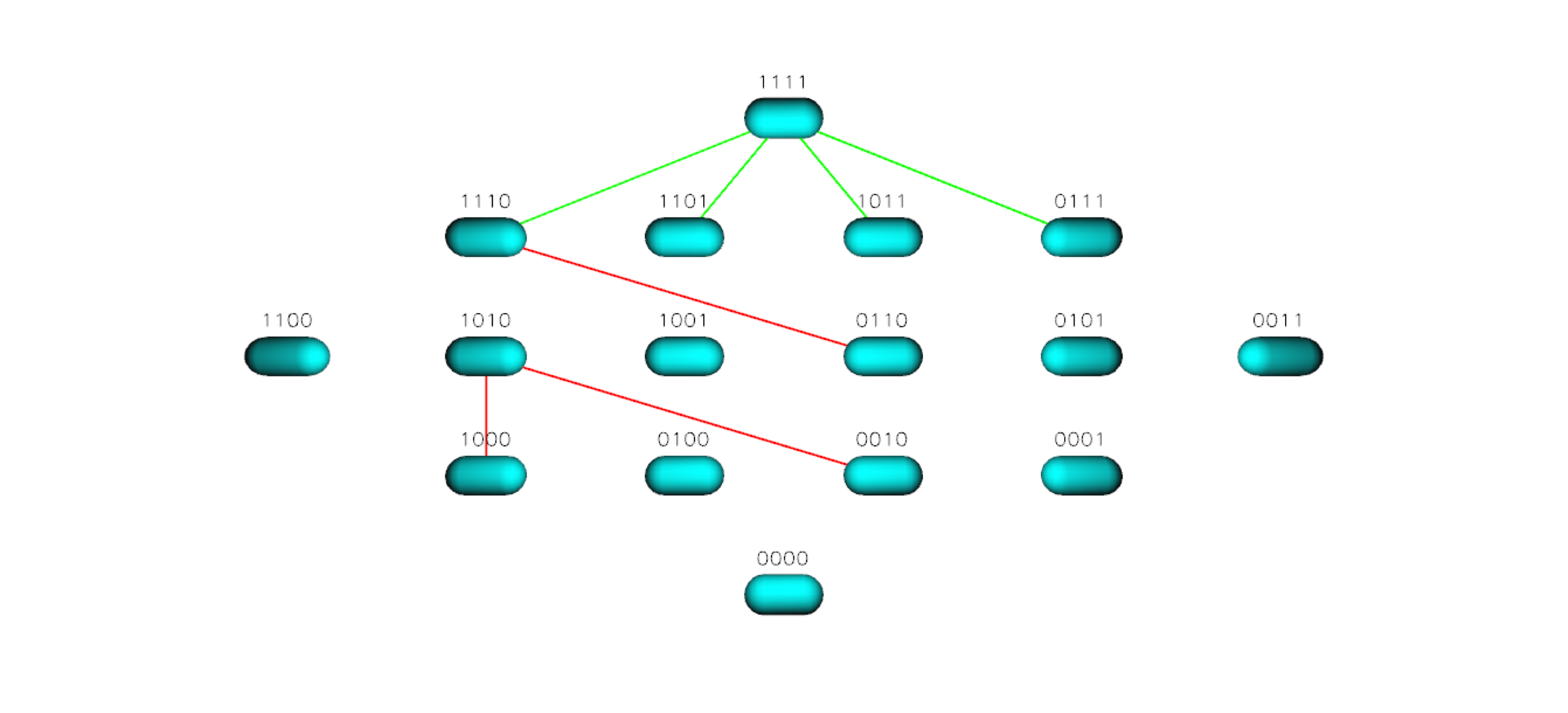


Figure S24 TEM 85 Landscape for Cefuroxime

Supplement: Figure S24 — Figures of TEM-85 Adaptive Landscapes. Ovals represent alleles. The names are given in binary code (See table 1). The absence of lines indicates no significant difference in resistance phenotypes. Green lines indicate an increase in resistance resulting from addition of a mutation. Red lines indicate an increase in resistance resulting from reversion. (DOCX) [file pone.0056040.s024.docx]

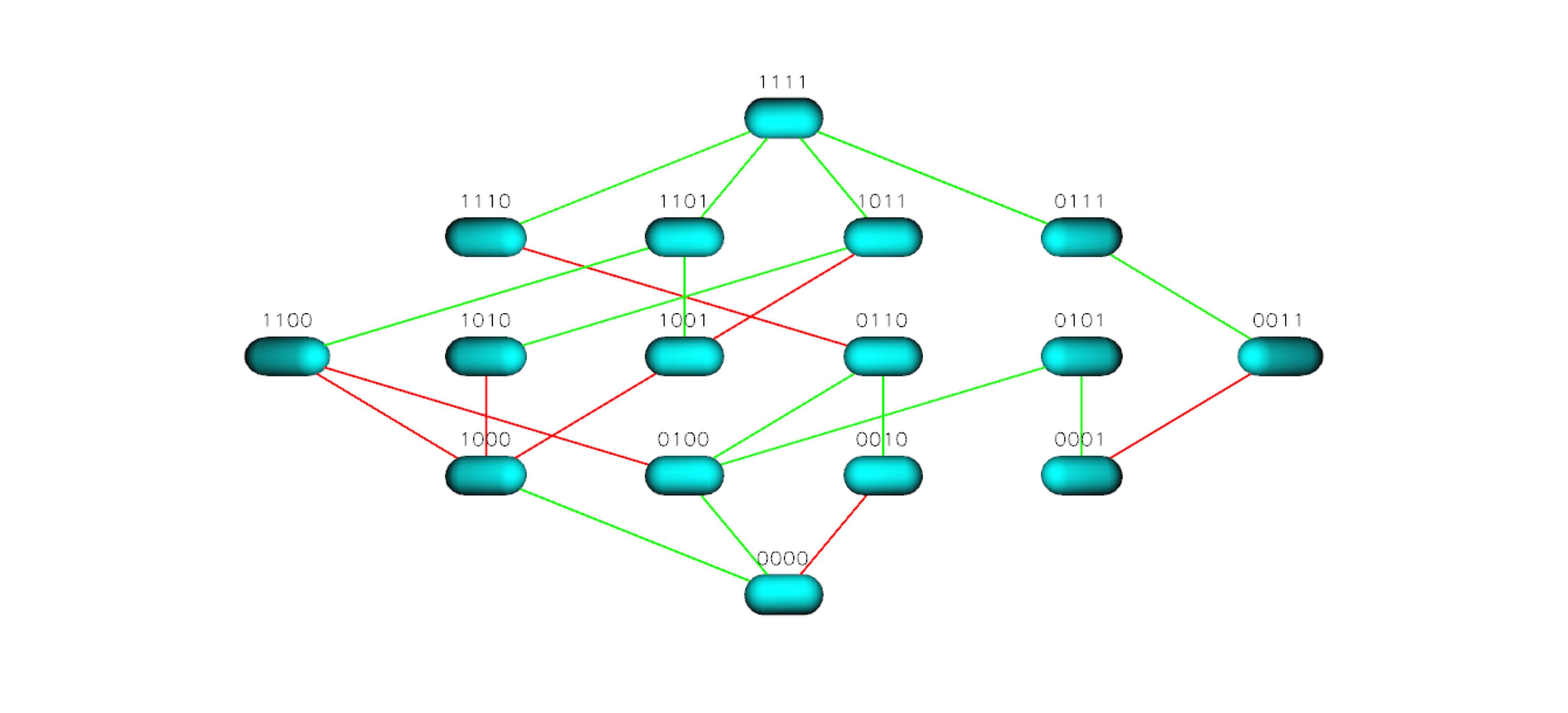


Figure S25 TEM 85 Landscape for Cefepime

Supplement: Figure S25 — Figures of TEM-85 Adaptive Landscapes. Ovals represent alleles. The names are given in binary code (See table 1). The absence of lines indicates no significant difference in resistance phenotypes. Green lines indicate an increase in resistance resulting from addition of a mutation. Red lines indicate an increase in resistance resulting from reversion. (DOCX) [file pone.0056040.s025.docx]

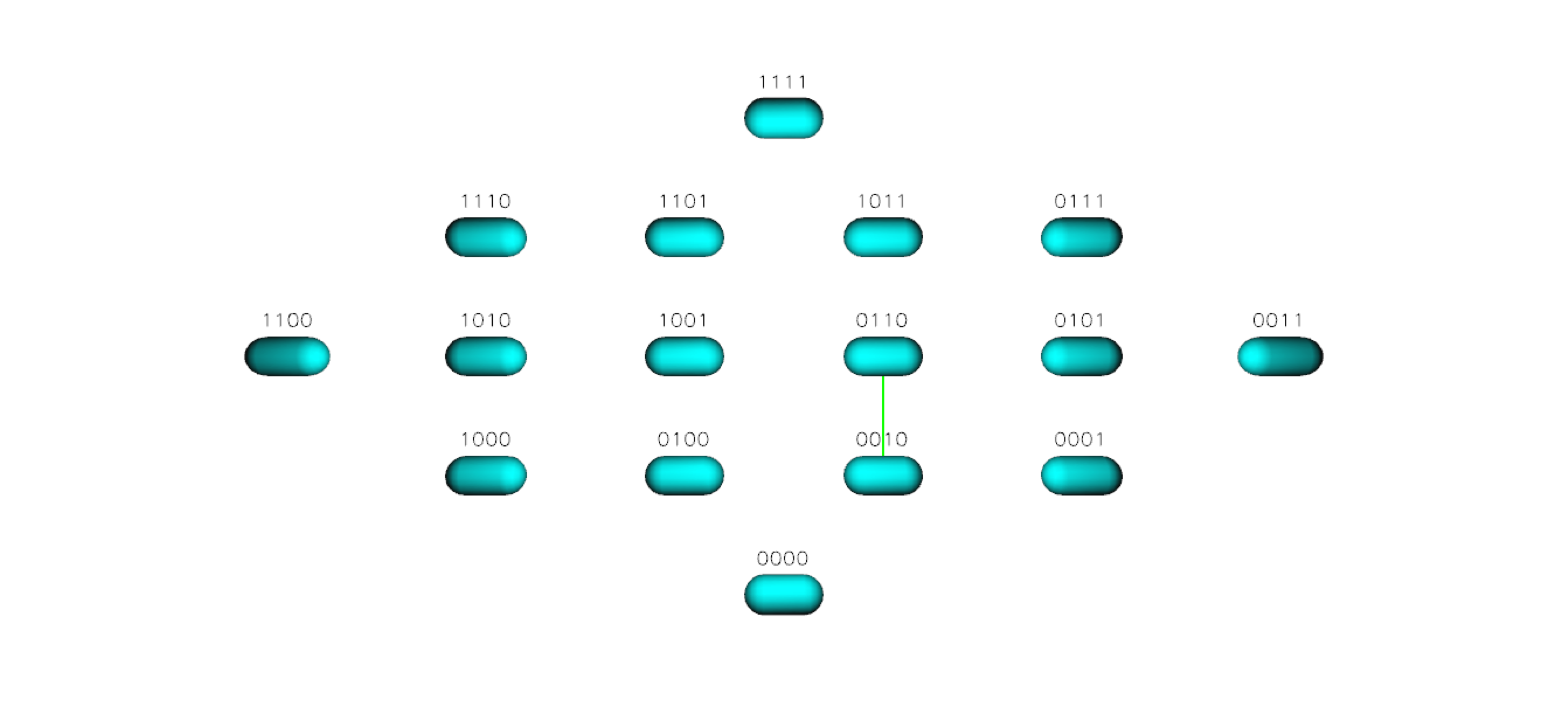


Figure S26 TEM 85 Landscape for Cefoxitin

Supplement: Figure S26 — Figures of TEM-85 Adaptive Landscapes. Ovals represent alleles. The names are given in binary code (See table 1). The absence of lines indicates no significant difference in resistance phenotypes. Green lines indicate an increase in resistance resulting from addition of a mutation. Red lines indicate an increase in resistance resulting from reversion. (DOCX) [file pone.0056040.s026.docx]

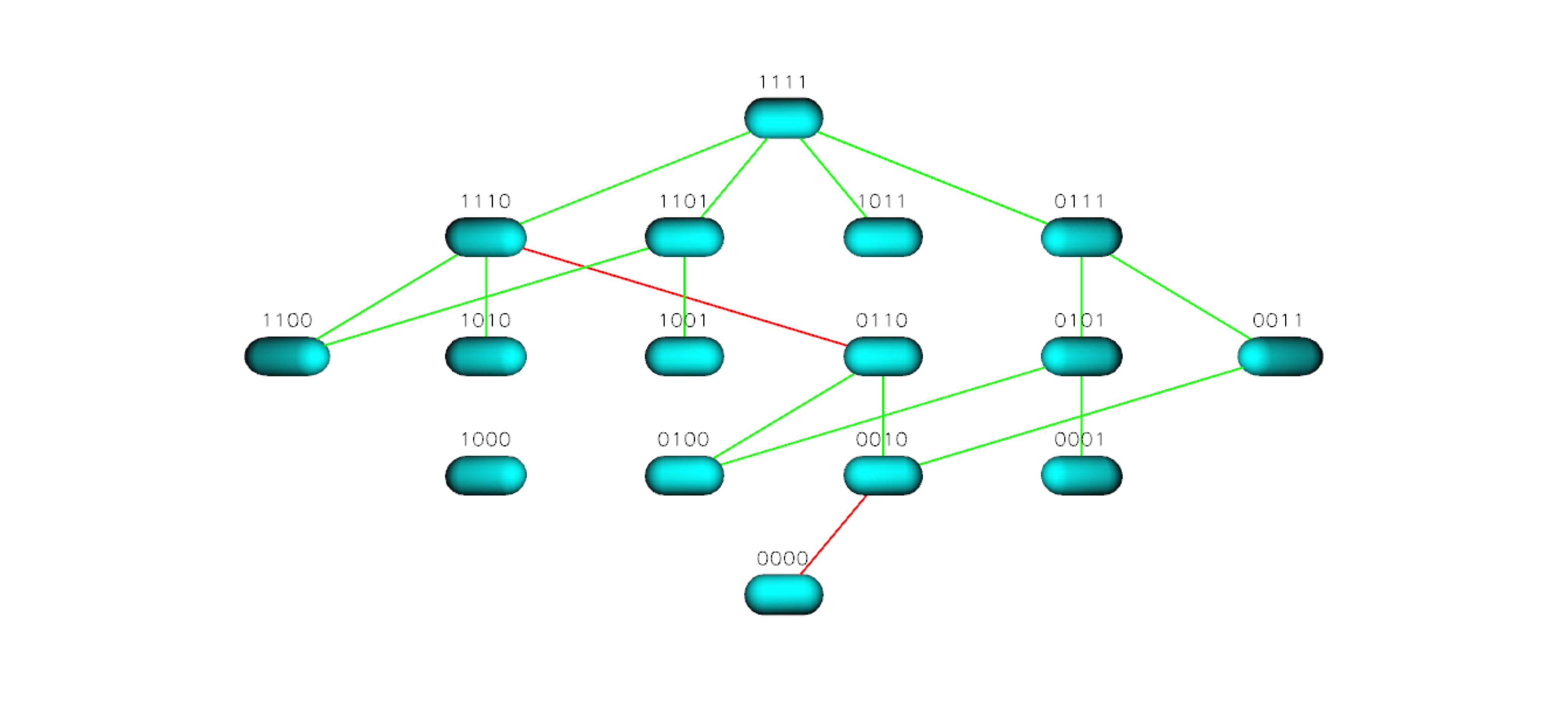


Figure S27 TEM 85 Landscape for Ceftizoxime

Supplement: Figure S27 — Figures of TEM-85 Adaptive Landscapes. Ovals represent alleles. The names are given in binary code (See table 1). The absence of lines indicates no significant difference in resistance phenotypes. Green lines indicate an increase in resistance resulting from addition of a mutation. Red lines indicate an increase in resistance resulting from reversion. (DOCX) [file pone.0056040.s027.docx]

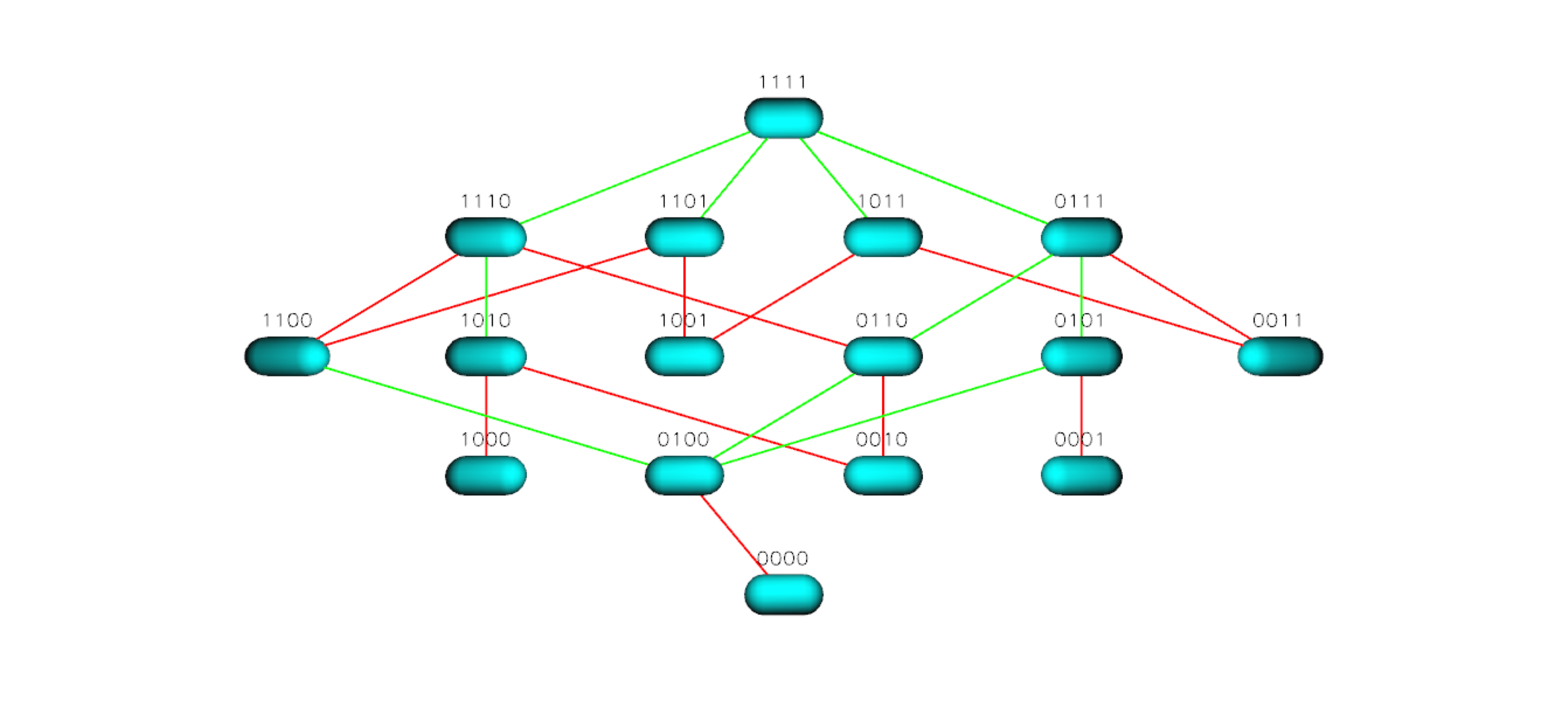


Figure S28 TEM 85 Landscape for Ampicillin + Sulbactam

Supplement: Figure S28 — Figures of TEM-85 Adaptive Landscapes. Ovals represent alleles. The names are given in binary code (See table 1). The absence of lines indicates no significant difference in resistance phenotypes. Green lines indicate an increase in resistance resulting from addition of a mutation. Red lines indicate an increase in resistance resulting from reversion. (DOCX) [file pone.0056040.s028.docx]

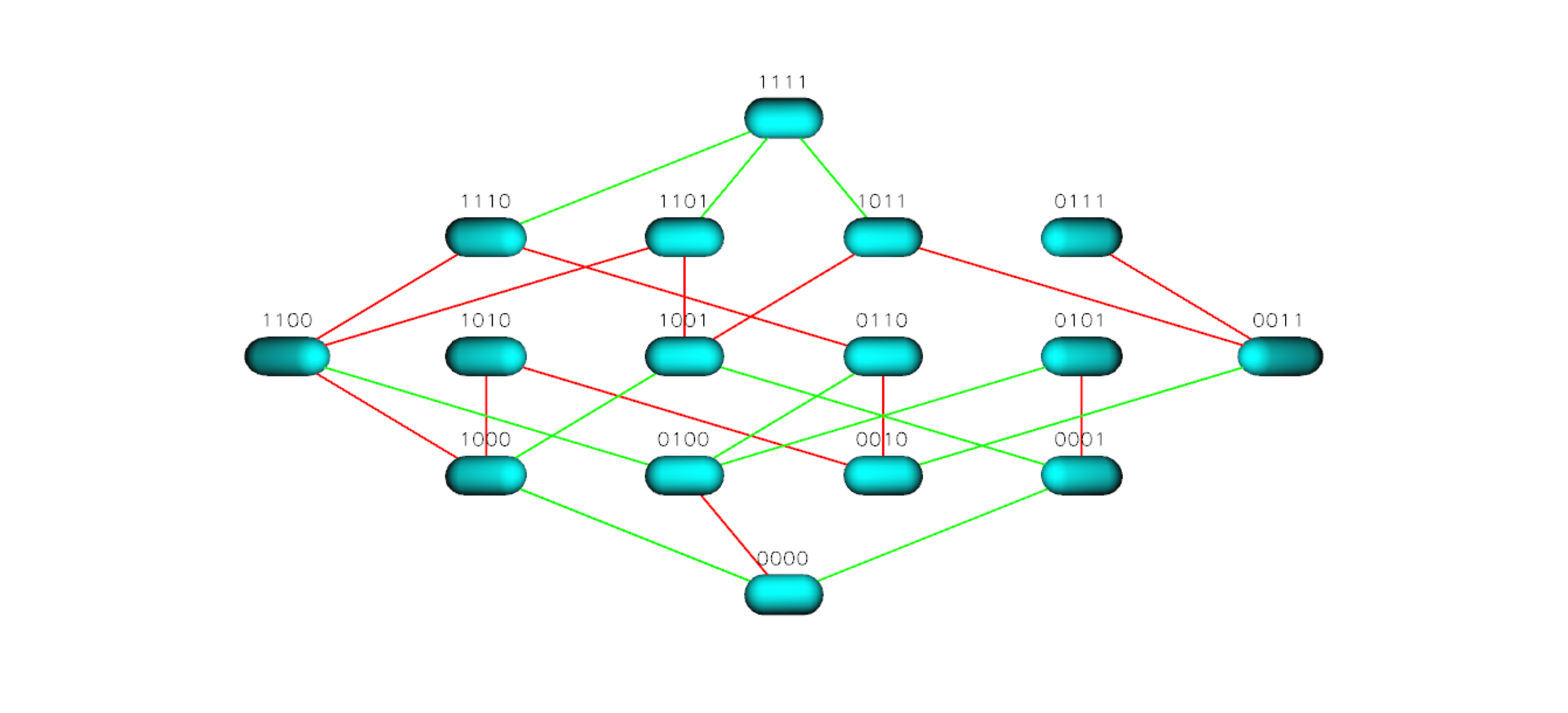


Figure S29 TEM 85 Landscape for Pipercillin + Tazobactam

Supplement: Figure S29 — Figures of TEM-85 Adaptive Landscapes. Ovals represent alleles. The names are given in binary code (See table 1). The absence of lines indicates no significant difference in resistance phenotypes. Green lines indicate an increase in resistance resulting from addition of a mutation. Red lines indicate an increase in resistance resulting from reversion. (DOCX) [file pone.0056040.s029.docx]

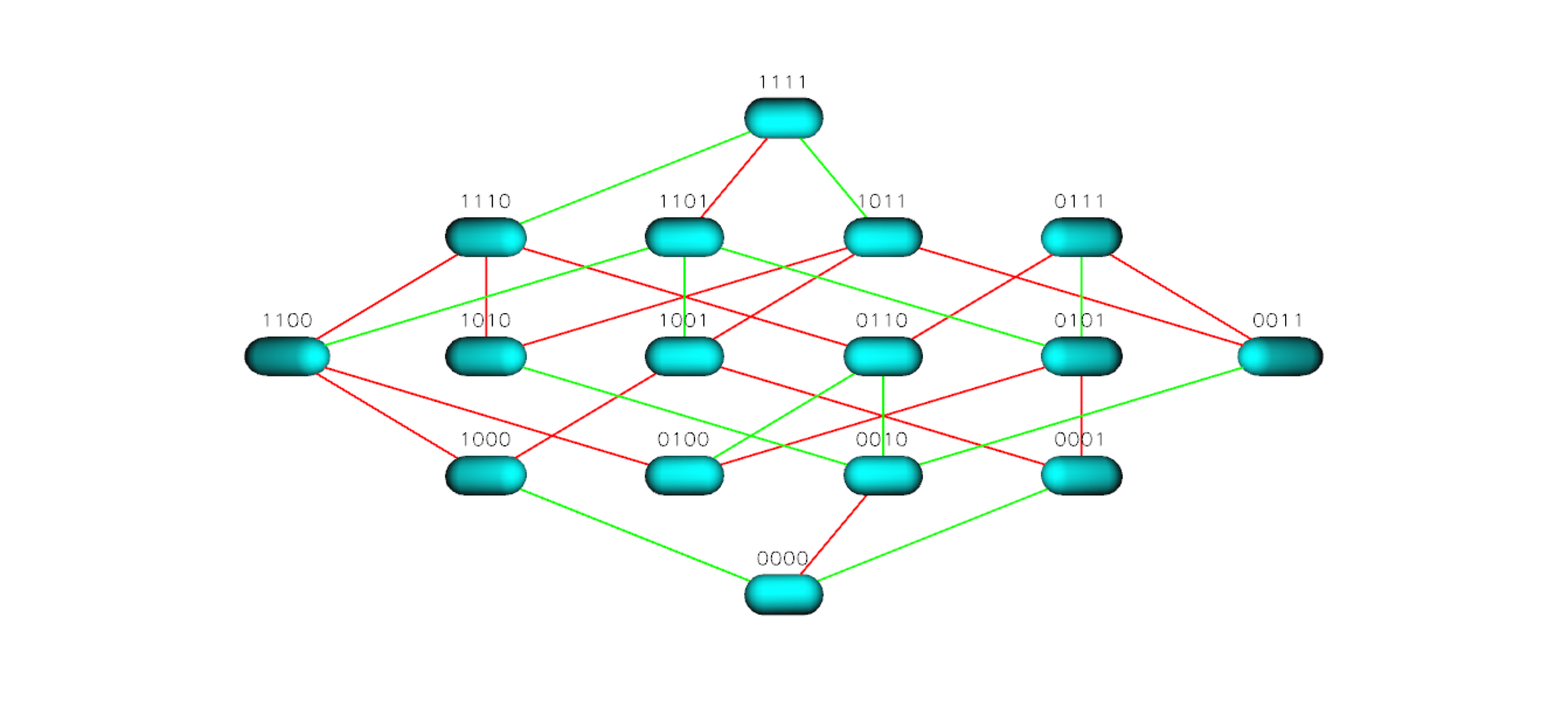
 Figure S30 TEM 85 Landscape for Amoxicillin + Clavulanate

Supplement: Figure S30 — Figures of TEM-85 Adaptive Landscapes. Ovals represent alleles. The names are given in binary code (See table 1). The absence of lines indicates no significant difference in resistance phenotypes. Green lines indicate an increase in resistance resulting from addition of a mutation. Red lines indicate an increase in resistance resulting from reversion. (DOCX) [file pone.0056040.s030.docx]

Figure S31


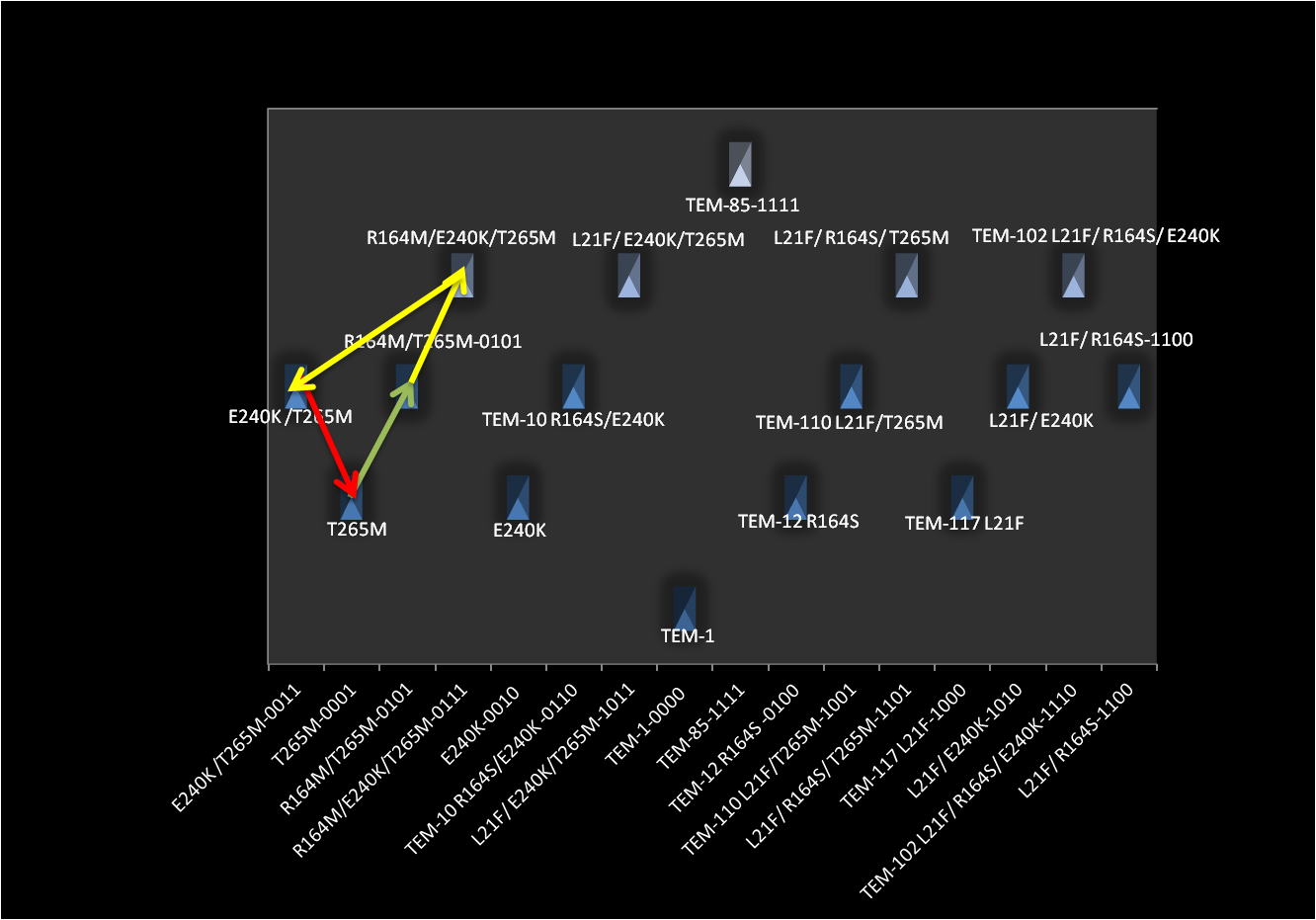

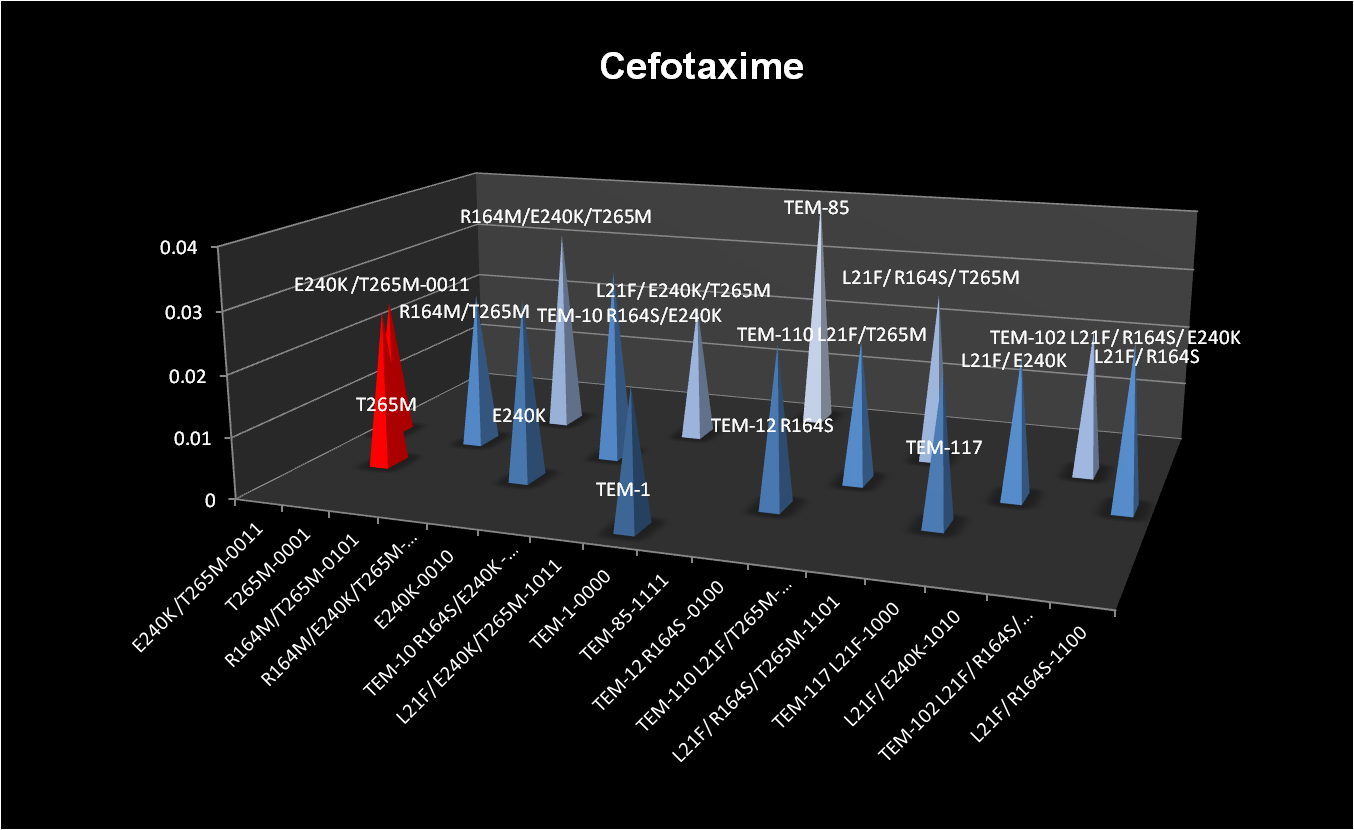

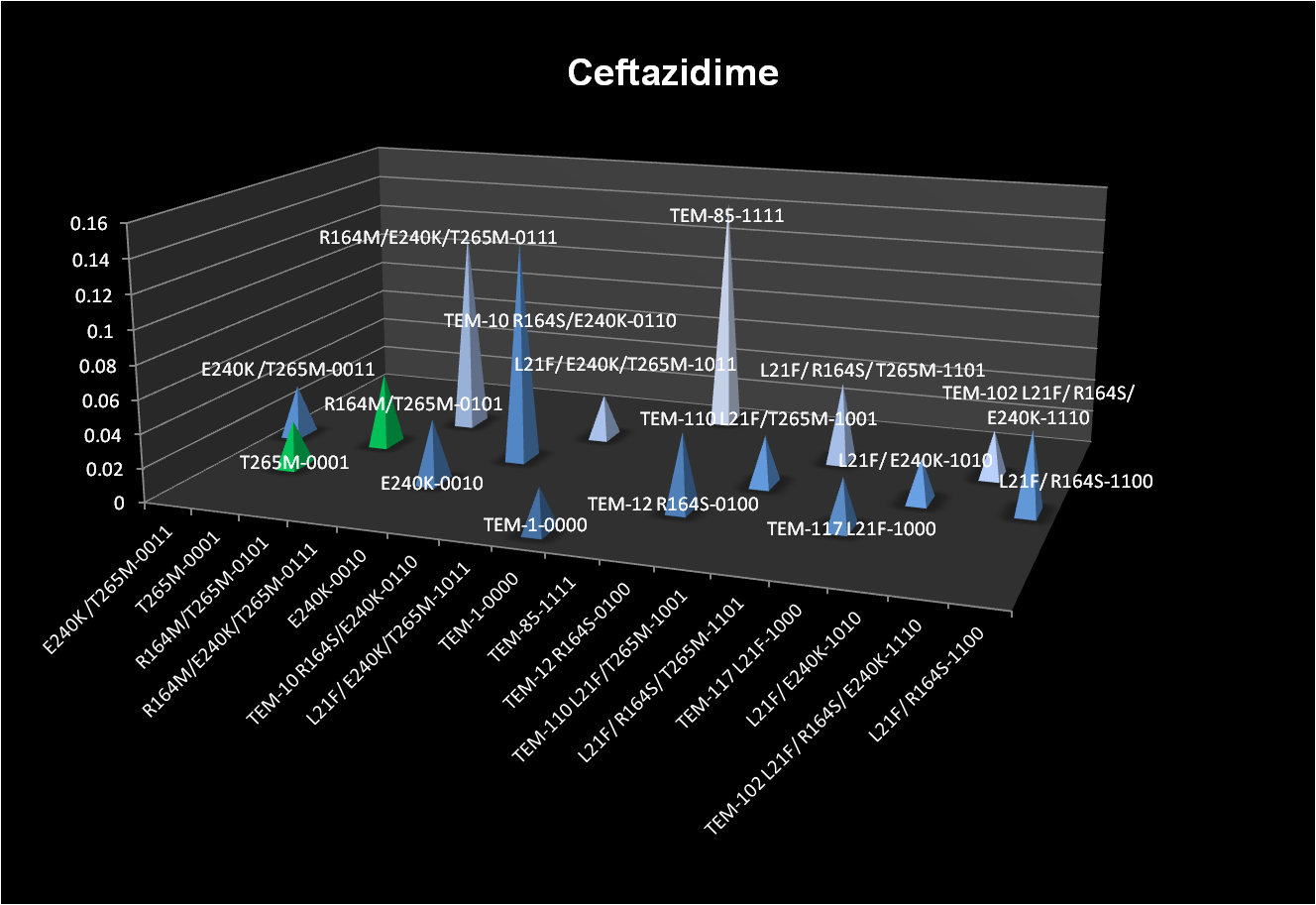

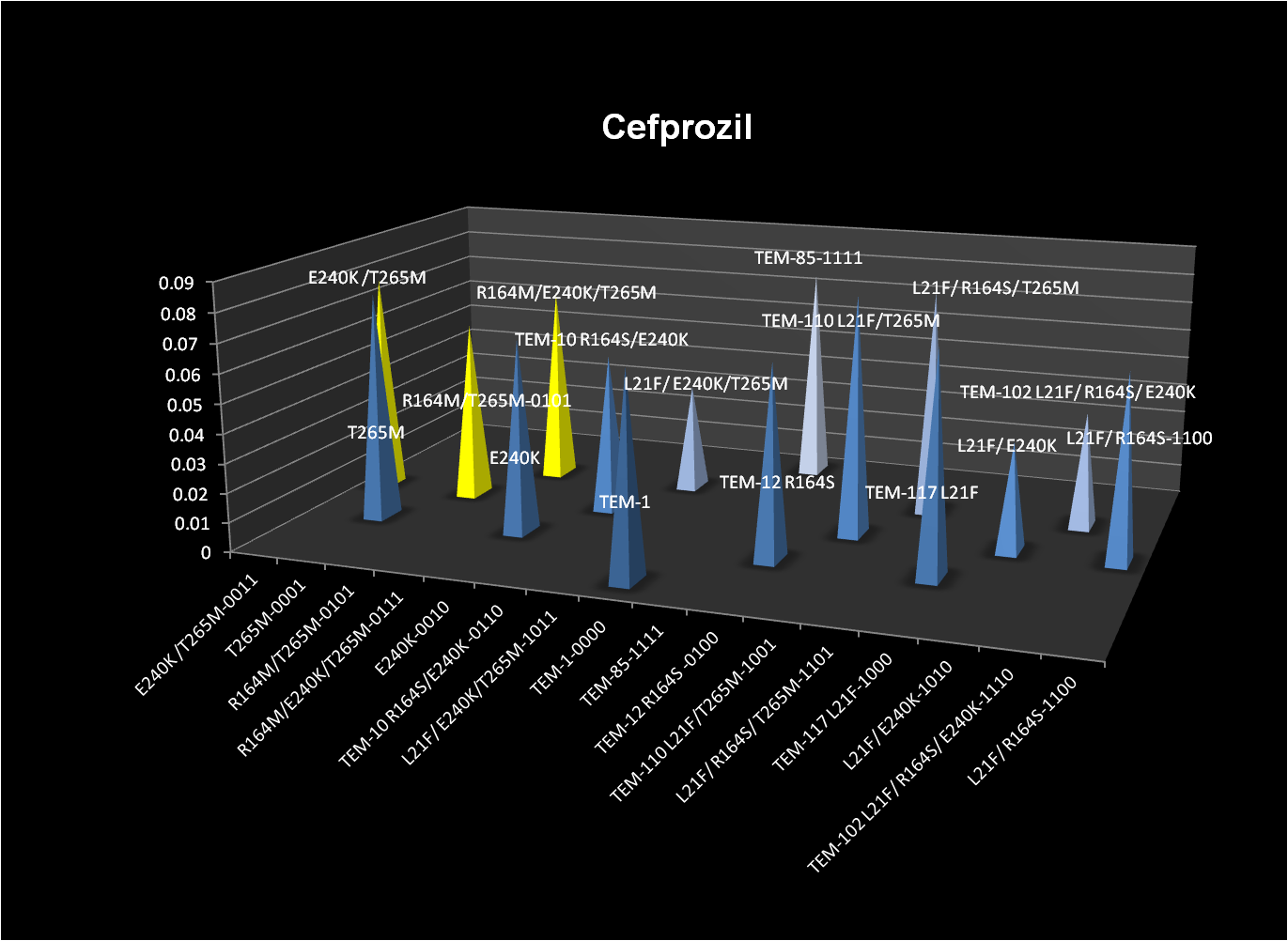

Supplement: Figure S31 — Example of one possible outcome from antibiotic cycling. a. (Top left) Composite cycle: The red arrow indicates an allele (T265M) that will be selected by cefotaxime. The green arrows indicate alleles that will be selected by ceftazidime. The yellow arrow indicates alleles that will be selected by cefprozil. b. (Top right) The TEM-85 adaptive landscape in cefotaxime. Red peaks indicate the adjacent alleles that are important during cefotaxime selection. c. (Bottom left) The TEM-85 adaptive landscape in ceftazidime. Green peaks indicate the adjacent alleles that are important during ceftazidime selection. d. (Bottom right) The TEM-85 adaptive landscape in cefprozil. Yellow peaks indicate the adjacent alleles that are important during cefprozil selection. (DOCX) [file pone.0056040.s031.docx]
